# Generative AI Chatbot for Diabetes Management: Formative 2-Part Qualitative Study Using DTalksBot Involving Patients and Clinicians

**DOI:** 10.2196/72553

**Published:** 2025-11-12

**Authors:** Soyun Jeon, Seolhee Lee, Esther Hehsun Kim, Jinsu Eun, Kwangwon Lee, Hajin Lim, Joonhwan Lee

**Affiliations:** 1 Department of Communication Seoul National University Seoul Republic of Korea; 2 Department of Transdisciplinary Studies Seoul National University Seoul Republic of Korea; 3 Interdisciplinary Program in Artificial Intelligence Seoul National University Seoul Republic of Korea

**Keywords:** generative AI chatbot, large language models (LLMs), retrieval-augmented generation (RAG), diabetes mellitus, diabetes management, online health information seeking

## Abstract

**Background:**

Diabetes mellitus requires continuous self-management to prevent complications. Patients frequently rely on online resources and mobile apps for diabetes-related information; however, these often lead to information overload, limited personalization, and difficulty in navigation. Generative artificial intelligence (AI) chatbots may address these challenges by providing accessible, personalized, and responsive guidance.

**Objective:**

This study aimed to explore the potential role of generative AI chatbots in diabetes management through a 2-part qualitative evaluation. Part 1 examined patients’ information needs, user experiences, and expectations. Part 2 investigated specialists’ perspectives on the practical utility of generative AI chatbots in supporting diabetes self-management. By incorporating perspectives from both patients and specialists, the study aimed to identify appropriate boundaries for the involvement of generative AI chatbots, reflecting the needs and expectations of both stakeholder groups.

**Methods:**

This study was conducted using DTalksBot, a generative AI chatbot powered by GPT-4 (OpenAI) and enhanced with retrieval-augmented generation. In Part 1, we aimed to understand the experiences, needs, and expectations of patients with diabetes. To achieve this, 24 participants engaged in structured chatbot sessions, completed postinteraction surveys, and participated in in-depth interviews. Data were analyzed using thematic and content analysis to identify patterns in user queries and experiences. In part 2, we invited 4 family medicine specialists to assess the accuracy of DTalksBot’s responses by reviewing conversation logs and to share expert insights on the future role of generative AI chatbots in diabetes management.

**Results:**

In part 1, a total of 24 patients submitted a total of 643 questions, which were categorized into 4 primary themes: personalized health advice and guidance (n=281, 44.6%), complications and comorbidities (n=174, 27.1%), medication and treatment exploration (n=111, 17.3%), and mental health management and support (n=30, 4.7%). Patients emphasized the advantages of generative AI chatbots over traditional information sources, including faster access to reliable content, reduced cognitive burden, and the ability to comfortably discuss sensitive topics. In part 2, specialists recognized the generative AI chatbots’ value in answering routine inquiries, but noted limitations in contextual accuracy, real-time data integration, and response personalization.

**Conclusions:**

Generative AI chatbots showed promise as complementary tools for diabetes self-management by offering accessible, reliable, and tailored support. This formative evaluation provides empirical evidence on how generative AI chatbots can address patient information needs and complement existing health care resources. To maximize utility, future generative AI chatbots need to integrate real-time health data, enhance contextual relevance, and align with clinical workflows to ensure safety, trust, and broader applicability.

## Introduction

### Background

Diabetes mellitus, a chronic condition caused by insufficient insulin production or usage [1-5], requires vigilant daily self-management to prevent complications such as cardiovascular disease, neuropathy, nephropathy, and retinopathy [1,3,6,7]. More than 85% of cases involve type 2 diabetes, which often improves with lifestyle modification, whereas type 1 diabetes usually requires a balanced diet and insulin therapy [2-5,8,9].

Because daily monitoring and lifestyle management occur largely outside clinical settings, individuals with diabetes require diverse and timely information [10-15]. They often search online more proactively than individuals with other chronic conditions, commonly using Google or Bing [16-18]. However, these search engines can overwhelm users with technical jargon, conflicting advice, and misinformation, highlighting the need for trustworthy digital support [18-21].

Most mobile apps and web-based platforms log quantitative self-monitoring data—blood glucose levels, dietary intake, and medication use—but rarely deliver personalized feedback or conversational support [9,12,22]. Their content is largely generic, and evidence for accuracy, clinical validity, and long-term benefit is limited [9,12]. Furthermore, the absence of real-time decision support has hindered adoption by both patients and clinicians [9]. Collectively, these limitations underscore the need for interactive and context-aware systems that provide individualized guidance.

To address these shortcomings, research on conversational interfaces has gained momentum [23-25]. Early rule-based chatbots, constrained by predefined scripts and decision trees, supported only narrowly defined tasks and offered limited flexibility or personalization, which reduced user engagement [23-28]. Recent advances in large language models have enabled generative artificial intelligence (AI) chatbots capable of interpreting complex queries and generating context-specific responses [29-34]. Systematic reviews have shown that such chatbots are being piloted across diverse health care domains, including chronic disease self-management, cancer care, and health behavior change. These systems often facilitate patient education, medication reminders, and emotional support, with generally high user acceptance but limited rigorous evidence of effectiveness [23,24,28]. Notably, a scoping review found that 30% of AI apps for chronic condition self-management focused on diabetes; however, most remain in early developmental stages and particularly lack support for emotional self-management, a critical component of comprehensive diabetes care [35]. Furthermore, a recent synthesis of conversational agents for chronic condition self-management highlighted that although such systems show promise in supporting patient engagement and self-care, their design and evaluation approaches are still characterized by conceptual fragmentation, indicating the need for more rigorous and multidisciplinary frameworks to enhance emotional and personalized support [36].

Beyond these design and evaluation challenges, unresolved safety risks further hinder the clinical deployment of generative AI chatbots. Insufficient transparency in training data and opaque inference mechanisms increase the risk of hallucinations—incorrect or misleading information presented as fact [31,37-45]. ChatGPT (OpenAI) and Gemini (Google) have provided incorrect guidance, particularly regarding medication-related inquiries, which underscores the need for cautious implementation and oversight in health care settings [37,46,47]. Consequently, some clinicians remain cautious about their use in clinical practice, whereas others view the technology as potentially viable if robust safeguards for safety and clinical reliability are implemented [31,44].

While interest in generative AI chatbots within health care is increasing, robust empirical studies for disease-specific implementations remain limited. Previous research has largely focused on general-purpose systems, leaving unanswered questions about whether generative AI chatbots can provide accurate, timely, and contextually relevant support to specific patient populations [48-54]. Furthermore, most existing studies assess either patient experience or clinician appraisal in isolation; accordingly, further research that integrates both stakeholder perspectives within a unified evaluative framework is warranted.

To address these gaps, we designed a 2-part qualitative study that incorporated both patient and specialist perspectives to explore the potential of generative AI chatbots in diabetes management. To ensure a safe and context-specific environment for interaction, we developed and used DTalksBot, a diabetes-specific generative AI chatbot powered by GPT-4 and enhanced with retrieval-augmented generation (RAG). By conducting interviews with both patients and specialists, we aimed to identify key insights into user perceptions, expectations, and the envisioned role of generative AI chatbots in diabetes management. The findings are expected to guide the safe and effective integration of generative AI chatbots into chronic disease care and provide actionable insights for clinicians considering AI-driven tools and for developers aiming to enhance chatbot design with improved contextual relevance, safety, and personalization.

### Objectives

A 2-part evaluation was conducted to explore how generative AI chatbots could support diabetes management through 5 research questions (RQs): RQs 1-4 were investigated in part 1 with individuals with diabetes, whereas RQ 5 was explored in part 2 with family medicine specialists.

In part 1, the following RQs were investigated. First, what types of questions do patients frequently ask, and what information do they seek? This question was examined by analyzing conversation logs between participants and DTalksBot to identify recurring question types and information needs. Second, how do patients evaluate the overall user experience of DTalksBot? This question was addressed through postinteraction surveys in which patients rated usability, message credibility, emotional support, empathy, trust, and overall satisfaction. Third, how does interacting with DTalksBot compare with patients’ use of online resources and consultations with health care providers? This question was explored by comparing interview narratives about generative AI chatbots with participants’ accounts of their typical online searches and clinician consultations. Fourth, what are patients’ expectations and preferences for generative AI chatbots in diabetes management? This question was investigated through thematic analysis of interview transcripts, focusing on desired functions and support features.

In part 2, the following RQ was explored: how do family medicine specialists evaluate DTalksBot’s responses, and what role do they envision for generative AI chatbots in diabetes management? This question was studied by analyzing interviews for perceived utility, limitations, and future directions.

## Methods

### Study Design

Previous studies have emphasized the importance of qualitative inquiry in understanding how individuals navigate and use online health information effectively [18]. In the context of chronic disease management, where patients face distinctive challenges in accessing, interpreting, and applying information, qualitative approaches are particularly effective in capturing the complexity of patients’ information needs and the contextual barriers they encounter [18]. Furthermore, as generative AI chatbots represent a novel and interactive medium for health communication, qualitative methods are especially suited to uncover the nuanced ways in which patients perceive, engage with, and form expectations of such systems. Therefore, this study adopted a qualitative research design to explore how generative AI chatbots could support diabetes management. Grounded in a constructivist paradigm, this approach enabled a deeper examination of how individuals with diabetes interact with generative AI chatbots and form expectations—insights that would be difficult to obtain through structured surveys or quantitative metrics.

In addition to qualitative methods, descriptive statistical analyses were also incorporated to summarize the overall user experience. Interviews and analysis were conducted by authors with expertise in human-computer interaction. None had previous relationships with participants. This study followed the Standards for Reporting Qualitative Research guidelines (Multimedia Appendix 1).

### Participants

For RQ 1 through RQ 4, we recruited Korean adult patients with diabetes who were actively managing their condition through medication, insulin injections, or regular blood glucose monitoring. This group was selected because individuals engaged in active self-management were more likely to seek health information online and exhibit proactive health behaviors [55,56]. Recruitment took place entirely online, outside of clinical settings, via nationwide diabetes-related community platforms. Interested individuals who viewed the recruitment notice submitted an application form, and the research team contacted them individually to confirm eligibility and arrange the study session. The sample size was determined based on existing guidelines for achieving data saturation in qualitative research [57].

While this study focused on Korean adults, the prevalence of diabetes in South Korea was comparable with that in many Western countries [5]. Although Korean patients with type 2 diabetes tended to have lower BMI than their Western counterparts, they exhibited high rates of insulin resistance and cardiometabolic complications—partly due to carbohydrate-rich diets and increased central adiposity despite normal weight [5]. Consequently, dietary regulation played a critical role in diabetes care, and patients could benefit from continued, personalized guidance. These population-specific characteristics underscore the relevance of generative AI chatbots as practical tools for daily self-management in the Korean context.

Participants included both patients with type 1 and type 2 diabetes, but no distinction was made during recruitment and analysis. This decision reflected the study’s focus on lifestyle-based self-management rather than disease-specific pathology. Type 1 diabetes generally begins earlier in life and requires lifelong insulin therapy due to autoimmune destruction of β-cells, whereas type 2 diabetes is associated with insulin resistance and lifestyle factors [1,3]. Despite these differences, both conditions required ongoing self-management, including blood glucose monitoring, dietary regulation, and physical activity [3]. Given these shared management needs, this study explored the potential of generative AI chatbots to support diabetes self-management in a broadly applicable context.

For RQ 5, Korean family medicine specialists with clinical expertise in treating diabetes were invited to provide expert evaluations. These participants were identified through professional referrals within the medical community. Similarly, a small number of expert participants were considered sufficient to yield meaningful insights, in line with previous recommendations for expert sampling in qualitative studies [57].

### Procedure

#### Overview

This study was conducted between September 2023 and June 2024, encompassing chatbot development, participant recruitment, and data collection and analysis. The study consisted of two parts: (1) patients interacted with DTalksBot and then completed an evaluation, and (2) specialists reviewed conversation logs and provided insights on the potential role of generative AI chatbots in diabetes management. Part 1 lasted 60 minutes, whereas part 2 lasted 90 minutes. This 2-part structure was designed to integrate insights from both patients and clinical experts to inform the future development of generative AI chatbots.

#### Part 1: Using and Assessing DTalksBot With Patients

Patients joined either in person or remotely and received a brief orientation. A presurvey collected demographic and health-related information, including diabetes type, duration, treatment methods, and typical sources of health information. The interaction with DTalksBot was divided into four 5-minute structured sessions (20 minutes total): session 1 allowed free exploration of diabetes-related topics; session 2 focused on daily management, including diet, exercise, and lifestyle habits; session 3 centered on medical treatment and emergencies; session 4 was open-ended for additional concerns.

Afterward, patients completed a postsurvey evaluating DTalksBot. Measures included usability [58], message credibility [59], perceived social support [60], empathy [61], trustworthiness [62], expectations [63], and satisfaction [64] (Multimedia Appendix 2). Follow-up semistructured interviews were then conducted to explore patients’ experiences and expectations regarding generative AI chatbots. These interviews explored how patients compared them with conventional online resources and clinical consultations and perceived the role of generative AI chatbots in diabetes self-management.

#### Part 2: Analyzing Log Data and Assessing DTalksBot With Family Medicine Specialists

In the second part, family medicine specialists reviewed conversation logs from the patient interactions and interacted with DTalksBot to evaluate its performance. They qualitatively assessed the accuracy, context relevance, and clinical appropriateness of its responses, as well as its ability to handle high-risk scenarios safely and to remain within the scope of verified, diabetes-specific information based on current clinical guidelines. They also identified specific areas requiring improvement. Subsequently, semistructured interviews were conducted using a guide informed by previous research [29], focusing on the broader role of generative AI chatbots in diabetes management. These expert insights informed both the evaluation of DTalksBot and broader considerations for designing and implementing generative AI chatbots in diabetes management.

### Developing the DTalksBot

Widely adopted generative AI chatbots, such as ChatGPT and Gemini, often exhibit hallucination and tend to deliver overly generalized responses that lack the specificity required for clinical use [31,38,45,65,66]. Although ongoing updates to large language models have reduced hallucination rates, these systems remain trained on potentially biased or unverified corpora, leaving unresolved challenges about data transparency and model explainability [25,31,45,67]. In high-stakes domains such as health care, where accuracy and contextual relevance are critical, these limitations can pose serious patient safety risks [31,37,38,43,44,46]. To mitigate these risks and ensure a safe experimental environment, we developed DTalksBot, a domain-specific generative AI chatbot specialized for diabetes management.

DTalksBot was developed by integrating GPT-4 with a RAG model to generate medically accurate and context-specific responses. While GPT-4 enabled natural language processing, the RAG model enhanced factual accuracy by retrieving information from a curated knowledge base. This database was built on validated sources, including the National Health Information Portal [68], the Korean Diabetes Association [69], and the Centers for Disease Control and Prevention [70], thereby ensuring evidence-based diabetes guidance [71].

The operational structure of DTalksBot was designed to maximize transparency and explainability while minimizing hallucinations. Reference materials were segmented into individual sentences and converted into vectors. When a user submitted a question, the system calculated the cosine similarity between the query and all sentence vectors, retrieved the top 5 most relevant sentences, and used GPT-4 to synthesize an accessible response. This design grounded each answer in verifiable sources and kept the reasoning traceable. By restricting outputs to verified diabetes-specific information and avoiding speculation, DTalksBot reduced hallucinations commonly observed in general-purpose generative AI chatbots [30,66,71-73].

**Figure 1:**
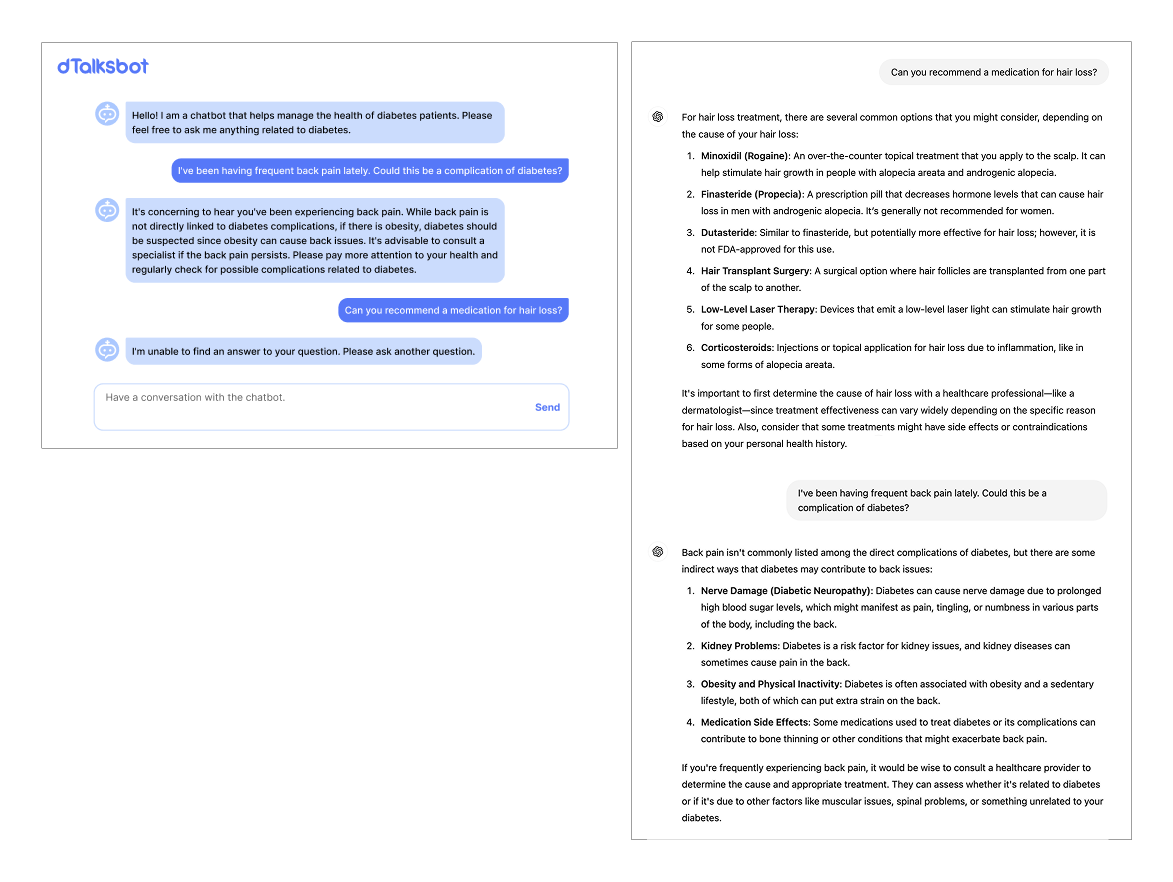
Example responses to patient queries about hair loss in the context of diabetes self-management. DTalksBot issues a safety-focused refusal (left), while ChatGPT provides a general answer (right).

Unlike general-purpose generative AI chatbots that answer any query, DTalksBot was restricted to diabetes-related topics, maintaining dialogue until the user chose to end the session and retaining previous conversational context to provide coherent follow-up responses. It declined to respond to unrelated queries—such as those about hair loss—thereby minimizing risk and maintaining thematic focus (Figure 1). When a question fell outside its curated knowledge base, DTalksBot prompted the user to pose an alternative question. The system prompt instructed DTalksBot to deliver concise yet supportive explanations with a brief rationale for each answer. It delivered condition-specific responses, such as dietary advice tailored to individuals with diabetes rather than generic public recommendations, and covered diverse aspects of diabetes management, including lifestyle modification, complication prevention, and general treatment guidance.

While DTalksBot provided comprehensive, evidence-based information, it intentionally excluded content on medication costs, insurance coverage, and investigational drugs in clinical trials, as these topics vary by individual circumstances and may pose safety concerns. It also did not offer medical diagnoses or prescriptions, instead directing users to seek clinician input for such needs. Furthermore, it did not integrate real-time biometric data, limiting its capacity for highly personalized recommendations. Nonetheless, its design bridged the gap between generic health information and the nuanced needs of patients with diabetes. This clinically bound scope-enabled patients and family medicine specialists to use the system in a safe, reliable, and clinically appropriate environment. Table 1 presents a comparison between DTalksBot and general-purpose generative AI chatbots. Example responses are provided in Multimedia Appendix 3.

**Table 1:** Key differences between general-purpose generative artificial intelligence chatbots (eg, ChatGPT and Gemini) and DTalksBot, a GPT-4–based chatbot with retrieval-augmented generation for diabetes management.

Feature	General-purpose generative AI^a^ chatbots (eg, ChatGPT and Gemini)	DTalksBot
Domain specificity	Covers all topics, including nonmedical	Limited to diabetes-related topics
Knowledge source	Trained on large-scale web data with limited source verification	Based on verified medical sources
Response and reasoning	No explicit reference to sources; reasoning unclear	RAG^b^ with validated sources and transparent reasoning
Personalization level	Generic advice for general users	Diabetes-specific advice
Safety for clinical use	Not clinically validated; may be inaccurate	Designed for safe diabetes management under clinical guidance

^a^AI: artificial intelligence.

^b^RAG: retrieval-augmented generation.

### Ethical Considerations

The research was conducted strictly following ethical guidelines and received approval from the Institutional Review Board of Seoul National University (IRB 2312/004-016). Informed consent was obtained from all participants after explaining the study’s purpose, procedures, and potential risks and benefits. Data were anonymized before analysis and securely stored. Each participant received 20,000 KRW (approximately US $15) as compensation.

### Data Analysis

For RQ 1, thematic and content analysis were used to examine DTalksBot interaction logs. Thematic analysis identified recurring question types, while content analysis quantified their frequency. Related questions were grouped using an affinity diagram to structure patients’ primary concerns. For RQ 2, descriptive statistics summarized user feedback. For RQ 3, thematic analysis of interviews explored how patients compared generative AI chatbots with traditional online information-seeking and clinician consultations. For RQ 4, thematic analysis examined patient expectations and preferences regarding the support provided by generative AI chatbots. For RQ 5, interviews with specialists were thematically analyzed to assess DTalksBot’s accuracy, usability, and clinical alignment, and to gather expert insights on the potential role of generative AI chatbots in diabetes management.

### Data Saturation

Data saturation was defined as the point at which no new codes or themes emerged during thematic analysis [74,75]. Previous studies suggested that saturation typically occurred within 12 to 20 interviews in relatively homogenous populations [57,75]. For the specialist interviews, we used purposive expert sampling, as even a small number of domain experts can yield rich qualitative insights [57].

The first, second, and third authors independently conducted open coding on an initial subset of transcripts to ensure analytic diversity. After comparing their code lists, they held consensus meetings to merge overlapping codes and resolve discrepancies, producing a preliminary codebook. This codebook was iteratively refined and applied to the entire data set; saturation was confirmed when 2 consecutive transcript batches produced no new codes.

## Results

### Participants’ Demographic and Clinical Characteristics

A total of 25 patients with diabetes were initially recruited for part 1 of the study; however, 1 participant (P16) withdrew, resulting in a final sample of 24 patients (6 males, 18 females). More than half of the patients were in their 20s and 30s, and most had type 2 diabetes (n=15, 62.5%). The majority had been diagnosed within 5 years (n=15, 62.5%). Treatment approaches included medication (n=13, 54.2%) and insulin therapy (n=8, 33.3%). Patient participant characteristics are summarized in Table 2.

**Table 2:** Demographic and clinical characteristics of 24 patient participants in part 1.

Characteristics			Participants, n (%)
**Age range (y)**			
	20-29	8 (33.33)	
	30-39	5 (20.83)	
	40-49	3 (12.5)	
	50-59	4 (16.67)	
	60-69	4 (16.67)	
**Sex**			
	Female	18 (75)	
	Male	6 (25)	
**Chronic conditions**			
	Diabetes	9 (37.5)	
	Diabetes and other	16 (66.67)	
**Types of diabetes**			
	Type 1	7 (29.17)	
	Type 2	15 (62.5)	
	Unknown	2 (8.33)	
**Duration of diabetes (y)**			
	<1	4 (16.67)	
	1-5	11 (45.83)	
	5-10	4 (16.67)	
	>10	5 (20.83)	
**Diabetes treatment**			
	Medication	13 (54.17)	
	Insulin injections	8 (33.33)	
	Medication and insulin injections	1 (4.17)	
	No treatment	2 (8.33)	

Most patients primarily relied on clinician consultations (n=19) and internet searches (n=18) for health information. Use of mobile health apps was less common (n=6), and only a few patients had previous experience with generative AI chatbots (n=3). These results are presented in Table 3.

**Table 3:** Health information-seeking behaviors and the use of generative artificial intelligence chatbots among 24 patient participants in part 1.

Category			Counts, n
**Usual sources of health information**			
	Clinician consultation	19	
	Internet search	18	
	Mobile health app	6	
**Use of generative AI^a^ chatbots for health information seeking**			
	Yes (used)	3	
	No (not used)	21	

^a^AI: artificial intelligence.

Additionally, 4 family medicine specialists participated in part 2 of the study (2 female, 2 male). Out of 4, 2 had more than 10 years of clinical experience. All were board-certified and specialized in diabetes care at academic medical centers. Specialist characteristics are detailed in Table 4.

**Table 4:** Demographic and clinical characteristics of 4 family medicine specialists in part 2.

Specialist	Age range (years)	Sex	Specialty	Clinical experience (y)
E1	30-39	Female	Family medicine	9
E2	40-49	Male	Family medicine	20
E3	30-39	Female	Family medicine	9
E4	40-49	Male	Family medicine	10

### Types of Questions and Information Needs Identified From DTalksBot Interactions (RQ 1)

#### Overview

A total of 643 questions were collected from 24 patients with diabetes*.* Using an affinity diagram, we identified common question types and organized them into 4 themes. Although sessions 1 and 4 were open-ended and sessions 2 and 3 focused on specific topics, many queries ultimately reflected core concerns in diabetes management. Patients often asked similar questions across sessions regardless of the prompt. For example, questions about complications or treatment options appeared in both guided and free-form sessions. This consistency suggested that patients’ information needs remained stable across contexts. Consequently, we integrated data from all 4 sessions for a more comprehensive analysis, thereby enabling inclusive thematic and content analysis and providing deeper insight into how patients prioritize aspects of diabetes self-management.

We identified four themes: (1) personalized health advice and guidance, including lifestyle and diet questions (n=281, 44.63%); (2) concerns about complications and comorbidities, reflecting proactive risk management (n=174, 27.06%); (3) medication and treatment exploration, covering conventional, alternative, and conflicting treatment advice (n=111, 17.26%); and (4) mental health management and support, addressing psychological and emotional aspects of diabetes (n=30, 4.67%). Representative questions and DTalksBot responses, organized by theme, are provided in Multimedia Appendix 4.

#### Theme 1: Personalized Health Advice and Guidance

Analysis of patient interactions with the DTalksBot revealed that patients frequently sought both general health advice and highly personalized guidance. Patients valued practical insights on daily self-management, such as dietary choices, exercise routines, and medication timing. Patients posed questions such as:

My blood sugar level slightly exceeds 220 after meals. Can you provide medical advice on this?

P8

I’ve been using an insulin pump for six years and my average blood sugar level has been stable at 6.3 for five years. Can I stop using the pump and switch to medication instead?

P3

These questions demonstrate that patients used generative AI chatbots not only for general knowledge but for responses tailored to their health profiles. By sharing personal health metrics, they sought feedback customized to their physiological conditions and daily routines, rather than receiving generic suggestions. This reflected a tendency to use generative AI chatbots as interactive tools for self-management, particularly when seeking guidance aligned with personal medical history and real-time data.

#### Theme 2: Concerns About Complications and Comorbidities

Many patients expressed concerns about diabetes-related complications and comorbidities. They frequently asked about the types, prevention, diagnosis, and treatment of complications. For instance:

How often should regular check-ups be conducted for managing complications?

P1

Several patients described symptoms and inquired whether they were related to complications. For example:

Is frequent eye redness related to diabetes complications?

P5

Sometimes my toes tingle. Is this related to my blood sugar levels?

P12

These questions illustrated that patients actively monitored bodily changes and sought to determine whether they indicated complications. This highlighted the role of generative AI chatbots in helping patients interpret symptoms and make informed self-management decisions.

In addition, some patients showed interest in how diabetes interacts with other chronic conditions. For instance:

Does hypertension affect blood sugar control? I'm wondering if treating hypertension will help with my diabetes.

P8

I have hepatitis B and diabetes. What complications should I be aware of when managing both conditions?

P5

These inquiries showed how patients with multiple chronic conditions seek guidance on how managing one condition affects another. This underscored the need for holistic, comorbidity-aware advice and highlighted the potential of generative AI chatbots to support proactive and whole-person diabetes care.

#### Theme 3: Medication and Treatment Exploration

Questions related to medication and treatment represented the third most frequently asked category, emphasizing patients’ active efforts to verify, compare, and interpret information about therapies and health metrics. This theme reflected patients’ interest in understanding the safety, efficacy, and relevance of various treatments—both medical and alternative—in the context of diabetes management. Many inquiries centered on the credibility of nontraditional or supplemental treatments. For example:

I heard that drinking a lot of corn water can completely cure type 1 diabetes. Is that true?

P17

Is it true that drinking vinegar water helps with blood sugar management?

P18

Patients often turned to DTalksBot to assess the validity of unverified claims encountered online or recommended by nonmedical sources. These questions illustrated how generative AI chatbots can serve as a trusted checkpoint for evaluating non–evidence-based information in everyday contexts.

Additionally, some patients expressed concerns about conflicting professional guidance. For instance:

My doctor recommended that I wear a continuous glucose monitor and advised me to keep my blood sugar level below 6.3. However, many doctors overseas consider a level up to 7.0 as normal. Should I follow my doctor’s recommendation of maintaining 6.3, or is 7.0 an acceptable target based on international standards?

P3

Such inquiries suggested that patients frequently encounter discrepancies in clinical recommendations and use generative AI chatbots to seek clarification. These interactions highlighted the potential of generative AI chatbots to mediate—not to override medical advice, but to help patients navigate uncertainty and make informed decisions amid conflicting opinions.

#### Theme 4: Mental Health Management and Support

The psychological and emotional burdens of managing diabetes were commonly reported, with many patients seeking strategies to maintain mental resilience and to cope with the unique stressors of their condition [76]. This theme illustrated a consistent need for mental health support as patients adjusted to lifestyle changes, dietary restrictions, and the broader impacts of diabetes on daily life.

Patients expressed curiosity about how daily stress affects blood sugar levels and overall diabetes outcomes. For example:

How does stress affect blood sugar levels?

P8

Can stress be a cause of diabetes?

P13

These questions reflected patients’ desire to understand how stress might influence the onset or progression of diabetes.

Beyond general curiosity, some patients expressed concern about the behavioral consequences of stress, including emotional eating and loss of motivation. For example:

When I’m stressed, I find myself craving sweets. Can you recommend effective stress-relief methods for diabetes patients?

P15

Such inquiries underscored the need for practical, empathetic guidance to manage stress-related behaviors.

Others sought more specific coping strategies tailored to their psychological challenges. For instance:

How can I best manage stress related to living with type 1 diabetes?

P14

Please provide specific stress management strategies for individuals with diabetes.

P23

These questions highlighted the long-term mental strain associated with chronic disease management and a demand for personalized mental health support. This aligned with the concept of individualized care discussed in theme 1 and reinforced the value of emotional guidance in digital health interventions.

### User Experience of DTalksBot (RQ 2)

Participants evaluated their experience with DTalksBot through a postinteraction survey using a 5-point Likert scale. Usability scored a mean of 4.06 (SD 0.78), reflecting that the system was generally easy to use. Message credibility received a mean of 4.08 (SD 0.86), suggesting that users trusted the accuracy of the information provided.

Emotional support was rated at 3.42 (SD 0.79), indicating a moderate level of perceived support, while empathy scored 3.08 (SD 0.93), reflecting a modest degree of emotional resonance. Trust in DTalksBot received a mean of 3.69 (SD 0.99), showing a moderate level of confidence in its performance.

Expectations were rated at 4.06 (SD 0.95), indicating generally high hopes for its utility. Satisfaction scored 3.94 (SD 0.88), suggesting that participants were largely satisfied with their experience. Overall, participants rated all dimensions above the midpoint, with relatively higher scores for usability, credibility, and expectations, and lower—but still positive—ratings for emotional support and empathy.

### Comparison of Chatbot Interactions With Online Resources and Health Care Providers (RQ 3)

#### Overview

This section examined how chatbot-based information-seeking compared with using search engines or online communities, and how generative AI chatbot interactions contrasted with in-person consultations with health care providers. We focused on how perceptions of immediacy, credibility, and emotional safety differed across these sources.

#### Comparison of Online Health Information Seeking

Patients traditionally relied on search engines like Google or online communities to obtain health information. While these sources provided a broad range of experiences and resources, they also presented notable limitations. First, unlike generative AI chatbots, search engines typically require users to spend considerable time sifting through extensive lists of results and assessing each source for relevance and accuracy. For example:

On portal sites, related content for the searched term appears in a long list, taking a lot of time to find the necessary information, but the chatbot provides immediate answers to questions.

P5

These insights highlighted the efficiency and accessibility of generative AI chatbots. By delivering curated responses instantly, generative AI chatbots could reduce the cognitive and time burden typically involved in online searches.

Second, while online communities offered insights grounded in lived experiences, participants often found user-generated content to be inconsistent and difficult to verify. For instance:

In online communities, many people share their blood sugar management know-how based on their experiences, so I’ve gained a lot of information there. But sometimes, it’s hard to tell if what someone says applies to everyone, so it would be helpful to verify it through the chatbot.

P10

This comparison suggested that generative AI chatbots were perceived as more dependable sources of information. Their consistent filtering of content helped minimize the effort required to evaluate diverse sources, positioning them as preferred tools for reliable guidance.

Additionally, the generative AI chatbots were appreciated for delivering condition-specific guidance. Since online portals predominantly catered to type 2 diabetes, patients with type 1 diabetes often felt underserved [3,8,9]. For instance:

There’s so much varied information on portals, but the chatbot only gives me diabetes-related information, which makes it feel more tailored to what I actually need.

P19

There’s so much information on Type 2 diabetes on the portal sites, but the chatbot gave me advice that actually fit my Type 1 needs.

P14

In summary, patients highlighted the limitations of traditional online resources, such as search engines and online communities, in their health information–seeking process. Search engines often required significant time and effort to filter through extensive and inconsistent content, while online communities provided unverified user perspectives. In contrast, the generative AI chatbots were valued for their ability to deliver accurate, professionally verified, and diabetes-specific information, offering a more efficient and reliable alternative tailored to individual needs.

#### Comparison of Conversations With Clinicians

Patients with diabetes typically visit clinicians every 2 to 6 months for consultations and medication prescriptions. Comparing these routine medical visits with interactions using generative AI chatbots revealed distinct strengths in each approach. About half of the participants noted little difference between the questions they asked clinicians and those posed to the generative AI chatbots, reporting similar responses. However, the other half identified distinct differences, especially regarding the complexity and personalization of their inquiries.

Patients often preferred generative AI chatbots to verify general information, clarify existing knowledge, or address questions they had forgotten to ask during consultations. Generative AI chatbots offered a more relaxed environment, allowing participants to ask questions freely without concerns about time constraints, social discomfort, or disrupting the clinicians’ schedule. For instance:

I can ask the chatbot any question, anytime, without worrying about the doctor's schedule or other waiting patients.

P15

Sometimes I get nervous and forget to ask questions during my consultation, so it’s nice to have the chatbot to ask follow-up questions I remember later.

P24

These insights suggested that patients viewed generative AI chatbots as valuable complements to medical visits, particularly for minor, follow-up, or lingering questions.

Patients also valued the freedom to raise sensitive or awkward questions that they might hesitate to raise with clinicians. For example:

Sometimes, due to time constraints, I can’t ask all my questions, and some feel a bit awkward to bring up directly. With the chatbot, I can ask about various topics freely, even those that might feel uncomfortable in person.

P20

This nonjudgmental setting encouraged more open discussions, suggesting that chatbots could address the everyday uncertainties common in chronic care.

In contrast, patients preferred to leave complex, data-specific inquiries, such as interpreting laboratory results, adjusting medication, and modifying insulin dosages, to their clinicians. These interactions required clinical expertise and access to personal medical histories. For example:

Since my doctor has all my blood sugar records and a history of my medications, I feel more comfortable asking specialized questions related to my specific data.

P20

This contrast highlighted a complementary role for generative AI chatbots in diabetes management. While generative AI chatbots offered ongoing, accessible support between visits, high-stakes decisions remain within the domain of clinicians.

Overall, these findings indicated that although generative AI chatbots could not replace professional medical advice, they might effectively support daily diabetes management. By providing reliable, immediate health information and reducing the cognitive load of self-management, generative AI chatbots could facilitate a more balanced and holistic approach—reinforcing rather than replacing the clinicians’ role.

### Desired Roles and Perceived Risks of Generative AI Chatbots (RQ 4)

#### Overview

The interviews explored patients’ expectations and desired roles for generative AI chatbots in diabetes management. As most patient participants had no previous experience with generative AI chatbots, this study offered a rare opportunity for direct, hands-on interaction with DTalksBot. Their feedback, grounded in actual interactions rather than theoretical concepts or superficial impressions, provided practical insights into both the potential roles and perceived limitations of these tools in diabetes management.

#### Evidence-Based and Reliable Responses

Patients consistently highlighted the value of generative AI chatbots as efficient tools for health information–seeking, emphasizing their ability to provide reliable, specific, and actionable information. Trustworthiness and accuracy emerged as critical features, particularly considering the overwhelming and often conflicting nature of diabetes-related content online. For example:

The most important thing for a chatbot is providing accurate information.

P7

It provides only the information that I need, offering specific and reliable responses.

P11

Patients clearly preferred generative AI chatbots that provided accurate, reliable, and tailored health information, and expressed that these qualities directly influenced their sense of trust.

Patients also emphasized the importance of evidence-based recommendations to enhance credibility. For instance:

If the chatbot could summarize recent treatments, medications, or research findings and provide the sources, it would significantly increase trust.

P1

This feedback underscored a critical demand for medical accuracy and source transparency, suggesting that generative AI chatbots needed to be anchored in up-to-date and verifiable information to be perceived as trustworthy tools.

#### Actionable and Personalized Guidance

Beyond general information-seeking, patients preferred practical guidance that aligned with their daily routines and real-world circumstances. For example:

The chatbot’s responses include easy-to-follow and tasty diet recommendations.

P5

The chatbot provided realistic management advice beyond what is typically found in books.

P6

Such comments reflected a desire for recommendations that could be easily integrated into daily life, particularly with respect to diet, exercise, and medication adherence.

A recurring theme in the interviews was the expectation for personalized and context-specific guidance. For example:

It would be great if the chatbot could understand my current health condition and provide personalized responses.

P4

These expectations highlighted the value of generative AI chatbots that went beyond generalized advice to deliver tailored and actionable guidance aligned with patients’ unique health conditions and self-management goals.

#### Limitations and Perceived Risks

However, patients also voiced concerns about potential risks, particularly in areas requiring critical health decisions. For example:

Discussions about adjusting medications based on data should probably be done with a physician.

P20

Diagnosing and treating complications like ketoacidosis requires precision and should involve a physician.

P22

These comments reflected patients’ pragmatic awareness of the limitations of AI tools. While generative AI chatbots could serve as supplementary resources, patients emphasized that they could not replace professional medical judgment, especially in situations that demand clinical expertise and high-stakes decision-making.

In summary, patients viewed generative AI chatbots as promising tools for efficient and accurate health information-seeking, particularly for clarifying complex or conflicting advice and delivering actionable guidance. However, they also recognized the importance of safeguards to ensure that generative AI chatbots served to complement rather than replace professional care, especially in contexts involving diagnostic accuracy and critical treatment decisions.

### Expert Perspectives on the Role and Limitations of Generative AI Chatbots in Diabetes Management (RQ 5)

#### Overview

This section presents insights from family medicine specialists who qualitatively assessed the clinical relevance, accuracy, and limitations of DTalksBot in the context of diabetes management. Additionally, their perspectives offered professional reflections on the potential roles of generative AI chatbots in supporting patients’ self-management and enhancing routine clinical workflows.

#### The Accuracy and Challenges of DTalksBot in Responding to Diabetes-Related Queries

DTalksBot was generally able to provide safe and appropriate guidance in high-risk scenarios requiring medical intervention. When asked about treatment adjustments, it consistently advised users to consult a clinician rather than provide a definitive answer. Specialists interpreted this approach as a strength, as it minimized the risk of misinformation and aligned with real-world clinical practice. Specialists noted that deferring to medical professionals in uncertain contexts reflects an appropriate and responsible strategy for health-focused generative AI chatbots.

For questions about diabetes medication, the chatbot consistently advised patients to consult their physician, which is the appropriate approach given the potential risks involved.

E1

However, DTalksBot lacked sufficient personalization in tailoring its responses to individual patient characteristics. While its dietary and lifestyle recommendations were generally evidence-based, specialists pointed out that it failed to consider comorbid conditions such as renal dysfunction or body weight variations that require modified guidance. For instance, although DTalksBot often encouraged a protein-rich diet, it failed to recognize the potential risks for those with kidney disease.

Typically, diabetic patients are advised to consume more protein than carbohydrates. However, for those with kidney dysfunction, protein intake must be restricted. The chatbot did not consider such individual variations.

E2

Finally, specialists observed that DTalksBot occasionally provided overly detailed or tangential information that reduced the clarity and relevance of its responses. This tendency was particularly evident when DTalksBot elaborated on loosely related topics. Such overexplaining was seen as potentially confusing for patients, as it introduced unnecessary complexity into otherwise straightforward inquiries. One expert recalled a case in which a question about corn silk water triggered an unrelated discussion about smoking and hypoglycemia.

The chatbot could have simply said ‘No’ to a question about the effects of corn silk water, but instead it brought up smoking and hypoglycemia, which made the answer more confusing.

E1

In summary, DTalksBot demonstrated a relatively high level of accuracy and a cautious approach to clinical questions. However, its limitations in personalization and contextual precision pointed to areas in need of further refinement. These findings underscore the importance of developing generative AI chatbots that not only provide accurate medical information but also adapt to the diverse needs and characteristics of individual patients.

#### Potential of Generative AI Chatbots in Diabetes Management

After reviewing DTalksBot’s interaction logs and using the system firsthand, the specialists also shared their views on how generative AI chatbots could be used in diabetes management. Specialists agreed that generative AI chatbots could play a meaningful role in supporting diabetes self-management by addressing routine questions and clarifying treatment-related information between medical visits. Emphasizing the importance of lifestyle management alongside medical treatments for patients with diabetes, E2 noted that continuous lifestyle adjustments are essential to effective diabetes care:

Lifestyle management is as crucial as medication for diabetes. A chatbot could provide ongoing support and answer questions between doctor visits, helping patients manage their condition more comfortably.

E2

This perspective suggested that generative AI chatbots could supplement routine care by offering consistent, reliable answers to questions about diet, exercise, and blood sugar control, thus reinforcing adherence to long-term management goals.

Furthermore, regarding alleviating provider workload, specialists viewed generative AI chatbots as useful for addressing lifestyle-related questions that might otherwise consume valuable consultation time. As E2 observed:

While we handle medication, the chatbot could manage lifestyle aspects like diet, creating a synergistic effect.

E2

This reflected a complementary division of roles, allowing clinicians to focus on clinical decisions while generative AI chatbots address everyday concerns. Such delineation could enhance consultation efficiency and promote better health outcomes through comprehensive and continuous support.

Additionally, specialists noted that generative AI chatbots could help organize patient inquiries and summarize their concerns to support continuity of care. As E1 suggested:

Many patients bring notebooks filled with questions they couldn’t address during their last visit, but if the chatbot could help keep track of these inquiries, it would help me as a doctor review and address these concerns in the next appointment.

E1

By identifying patterns in patient queries, generative AI chatbots could support a more cohesive approach to care and could enrich consultations by offering patients and clinicians a clearer understanding of ongoing needs and challenges.

#### Challenges of Generative AI Chatbots in Diabetes Management

While specialists were cautiously optimistic about the generative AI chatbots’ potential to enhance patient engagement, they noted that long-term behavior change—especially in areas such as diet and exercise—remains a significant challenge. E2 and E3 remarked:

Changing behavior through advice is difficult; thus, psychological well-being might be the main benefit of the chatbot.

E2

Behavior change through a chatbot is difficult. However, if the chatbot assists with practical tasks like scheduling doctor appointments or medication reminders, it could build rapport and aid in cognitive-behavioral therapy.

E3

These insights suggested that the primary role of generative AI chatbots might lie in maintaining motivation and supporting psychological well-being, rather than directly driving behavioral change. Future iterations could benefit from incorporating features that prioritize motivational support to foster long-term health engagement.

Specialists also noted limitations in the ability of generative AI chatbots to provide personalized advice. Due to the absence of patient-specific data, generative AI chatbots were not able to consistently account for critical variables like age, comorbidities, or treatment history, which were essential to individualized diabetes management. E3 highlighted this limitation, stating:

If an elderly patient uses the chatbot and it consistently advises that an average blood sugar level of 6.5 is normal, this could be dangerous. Important variables like age should be considered when providing health advice.

E3

These challenges suggested that while generative AI chatbots hold promise, further refinement was necessary for them to serve as effective tools for personalized diabetes management. Enhanced capabilities to interpret individual health metrics and respond to condition-specific factors could improve their utility across diverse patient populations.

Finally, specialists emphasized the importance of transparent data sources and explainable responses. They proposed that chatbots rely on curated, verified databases to ensure reliability and relevance in clinical contexts. As E4 commented:

ChatGPT has improved and provides accurate answers, but we don't know the data it was trained on, posing a risk of incorrect answers. The chatbot should be limited to a verified database like DTalksBot to ensure relevance and accuracy in a domestic context.

E4

This insight underscored a critical direction for future development—enhancing transparency and data fidelity to foster patient trust and encourage integration of generative AI chatbots into routine diabetes management.

## Discussion

### Principal Results

This study explored how generative AI chatbots could support diabetes management by addressing 5 key RQs: identifying the types of questions patients frequently ask and the information they seek (RQ 1), evaluating patients’ experiences with DTalksBot (RQ 2), comparing interactions with generative AI chatbots to online health information seeking and clinical consultations (RQ 3), investigating patients’ expectations for the use of generative AI chatbots in diabetes management (RQ 4), and analyzing specialists’ evaluations of DTalksBot and their perspectives on the broader role of generative AI chatbots in diabetes management (RQ 5).

As a part of RQ 1, we identified four primary themes reflecting the diverse needs of diabetes patients: (1) personalized health advice and guidance, (2) concerns about complications and comorbidities, (3) medication and treatment exploration, and (4) mental health management and support. These findings align with previous research, indicating that diabetes patients frequently seek online information about diet, exercise, complications, and medication [14,53]. In other words, the topics commonly explored through online searches also emerged frequently in DTalksBot interactions. However, patients tended to request more personalized information and emotional support from generative AI chatbots than from traditional online health-seeking behavior. Accordingly, future generative AI chatbots may benefit from incorporating not only personalized guidance and complication-related information but also stress management support as an integral component of diabetes management.

For RQ 2, patients generally perceived DTalksBot as usable, credible, and satisfying. High usability scores suggested that diabetes patients found the interface intuitive and accessible [27,28]. Strong message credibility further underscored the DTalksBot’s ability to deliver reliable information, reinforcing participants’ willingness to engage. While emotional support and empathy scores were moderate, this reflects a broader challenge for generative AI chatbots: difficulty in providing human-like empathy in health care contexts [32]. Nonetheless, enhancing empathetic conversation design could significantly influence chronic disease self-management behaviors [28].

For RQ 3, patients identified 2 key advantages of generative AI chatbots over traditional sources: mitigating information overload and providing a judgment-free environment. Patients described DTalksBot’s responses as not only trustworthy—grounded in validated sources through its RAG-based architecture—but also practical and actionable, enabling them to apply health guidance immediately in their daily routines. This combination of immediacy, safety, and utility illustrates how generative AI chatbots, when built on reliable data and domain-specific design, can effectively reduce the cognitive burden associated with navigating fragmented and inconsistent online health resources [24]. Furthermore, patients felt more comfortable asking sensitive or awkward questions—such as those related to unverified folk remedies—which they might hesitate to raise with clinicians due to fear of embarrassment. This aligns with theme 3 in RQ 1, where several patients asked about alternative or nonstandard treatments—topics they perceived as inappropriate for clinical settings but freely discussed with the chatbot. Such open dialogue reinforces the value of generative AI chatbots as complementary tools to formal care by fostering patient empowerment and supporting holistic chronic disease management. This finding also echoes previous research showing that chatbots promote open disclosure by removing social barriers to communication [29,53,77].

In RQ 4, 2 key insights emerged. First, participants emphasized the need for transparent and evidence-based responses, suggesting that citing medical guidelines or recent research could enhance credibility. Second, patients emphasized a preference for practical, personalized guidance, especially for meal planning and blood sugar management, reinforcing the value of user-centered and context-specific recommendations. Nevertheless, patients remained cautious about relying on generative AI chatbots for critical health decisions. This underscores the need to clearly define the role of generative AI chatbots as adjuncts to—not replacements for—professional care, ensuring that complex decisions stay under clinician supervision.

In RQ 5, family medicine specialists highlighted the complementary role of generative AI chatbots in diabetes care. They noted that generative AI chatbots could handle routine lifestyle queries, freeing clinicians to prioritize complex clinical decisions. Specialists also appreciated the potential of generative AI chatbots to bridge communication gaps, enabling patients to revisit concerns outside formal consultations. However, limitations included insufficient personalization for comorbid patients and the absence of detailed emergency guidance. Experts further recommended that future generative AI chatbots proactively seek clarification for vague queries and direct users to authoritative resources when necessary.

Overall, both patients and specialists recognized generative AI chatbots as valuable adjuncts in daily diabetes management. Patients envisioned on-demand support for daily self-management, including diet, glucose control, and emotional well-being, while specialists underscored the potential of generative AI chatbots to enhance consultation efficiency by addressing routine lifestyle queries. Importantly, patients valued the ability to deliver curated and trustworthy information more efficiently than traditional online sources, emphasizing the importance of reliable data in AI-driven health communication. Nevertheless, both stakeholder groups agreed that the generative AI chatbots are not intended to replace professional medical judgment or individualized clinical decision-making.

While patients expressed a strong desire for individualized, real-time guidance, specialists raised concerns about the risks of unsupervised hyperpersonalization—particularly in complex or ambiguous clinical contexts. These diverging perspectives underscore the need to define the appropriate boundaries of chatbot involvement. Rather than seeking to maximize chatbot autonomy, this study focused on identifying the types of support that can be safely handled by AI and those that require professional oversight. Conducted with individuals managing diabetes, this study not only demonstrates the feasibility of using generative AI chatbots in everyday health management but also offers foundational insights into the appropriate and inappropriate roles of generative AI chatbots in the context of patient-centered care. In doing so, it contributes to the early empirical groundwork necessary for establishing safe, effective, and ethically aligned roles for AI systems in chronic disease management.

### Future Directions

Integrating generative AI chatbots into routine health care offers a promising opportunity to improve accessibility by providing timely, tailored, and reliable support. Although this study centers on diabetes, the proposed directions may be applicable to a broader range of chronic diseases requiring ongoing behavioral guidance. Drawing from this study’s findings, we outline several design and research directions to enhance personalization, trustworthiness, emotional engagement, and clinical integration.

First, our findings demonstrated that domain-specific generative AI chatbots can mitigate many of the risks associated with opaque inference mechanisms and biased training data that characterize general-purpose systems [31,37-45]. As observed with DTalksBot, grounding responses exclusively in a curated, evidence-based knowledge base from verified medical sources enabled transparent reasoning and traceable information flow. This design minimized the potential for systematic bias and reduced the likelihood of disseminating inaccurate or misleading medical advice, a known concern in widely used chatbots such as ChatGPT and Gemini [37,46,47]. By restricting outputs to validated diabetes-specific content and clearly declining unsupported queries, DTalksBot illustrates how transparency in data provenance and scope control can enhance both safety and trustworthiness in AI-driven health communication.

Second, enhancing personalization through structured data integration may serve as a foundational design strategy. To tailor responses more effectively, future systems may benefit from integration with electronic health records (EHRs) and continuous glucose monitoring (CGM) data [78]. These integrations would enable generative AI chatbots to deliver personalized recommendations informed by historical health data. For instance, linking CGM data could enable chatbots to anticipate glucose fluctuations and offer proactive behavioral guidance, such as dietary and activity suggestions. Such features could be particularly beneficial for daily self-management, especially for patients with intensive insulin regimens.

While such data integrations enhance personalization, they also raise critical concerns regarding data privacy and user trust, which must be carefully addressed in future system design. Privacy-related behaviors are often shaped by privacy calculus theory, which posits that individuals weigh perceived benefits against risks when deciding whether to share personal data [79]. In practice, this can result in privacy paradoxes, where users express concern but still disclose data when they perceive high utility [80]. These dynamics suggest that trust and perceived control may influence user behavior more than technical safeguards alone. Accordingly, chatbot systems should prioritize not only data security, but also designs that promote user agency. One promising direction is the evaluation of data governance models—such as centralized versus decentralized architectures—that offer distinct trade-offs in transparency, control, and systemic risk [81].

Building on these considerations, caution is warranted regarding the extent of personalization and the privacy implications of data integration. While tailored recommendations can enhance user engagement, hyperpersonalized advice may pose clinical and regulatory risks. Instead, chatbots could provide guidance tailored to broader user groups (eg, age or comorbidity profiles) to balance relevance and safety. Additionally, compliance with regulations such as the Health Insurance Portability and Accountability Act in the United States and the General Data Protection Regulation in the European Union necessitates secure, transparent, and consent-driven data-sharing frameworks [82].

Third, ensuring the credibility and contemporaneity of chatbot responses requires robust information management and knowledge updating mechanisms. Advanced natural language processing and RAG models may enhance the chatbot’s ability to interpret user input and generate relevant responses [71,72]. Future generative AI chatbots will need to integrate diverse health care databases encompassing diabetes and comorbidities, thereby supporting holistic assessments [83]. Additionally, dynamic learning architectures are necessary to ensure content accuracy. Continuous updates from verified medical sources, including treatment protocols and new research findings, could help maintain chatbot relevance [30,66,84]. Ideally, these updates would be validated through expert-reviewed backend systems [85].

Fourth, addressing the emotional and cognitive needs of patients is essential in chronic disease management. Incorporating psychological models into generative AI chatbot design could enhance emotional support and behavioral guidance. Techniques based on cognitive behavioral therapy, combined with sentiment analysis, could allow chatbots to recognize and respond empathetically to users’ affective states [86,87]. While participants perceived the chatbot as moderately empathic, integrating such models could strengthen the chatbot not only as an information provider but also as a psychologically supportive companion [87].

Finally, generative AI chatbots are best positioned as collaborators within clinical workflows rather than autonomous decision-makers. Generative AI chatbots could alleviate the workload of health care providers by addressing routine patient inquiries, enabling clinicians to focus on more complex clinical decisions. Additionally, generative AI chatbots could act as intermediaries, collecting and summarizing patient inquiries between visits to improve consultation efficiency. Over time, generative AI chatbots may evolve into tools for long-term health management, tracking engagement, adherence, and health patterns while providing actionable insights [88]. To realize this potential, it is essential to establish clearly defined frameworks for human-AI collaboration—particularly in chronic care settings, where continuity and coordination are critical. To fully integrate chatbots into clinical workflows, future research may benefit from defining appropriate scope boundaries—clarifying which types of user queries chatbots can safely address, and when they should defer to human clinicians. Establishing such thresholds is essential to ensure patient safety and preserve the integrity of professional care.

### Limitations

Several limitations should be acknowledged. First, DTalksBot generated recommendations without access to real-time patient data. While it delivered general advice, future iterations may benefit from integrating real-time CGM data or EHR information to provide context-aware and patient-specific guidance.

Second, this study did not distinguish between type 1 and type 2 diabetes. Because these 2 groups differ in etiology, typical age of onset, and treatment requirements [1,3], subgroup analyses are needed to identify distinct information needs and interaction patterns.

Third, this study provides a cross-sectional perspective, which may not capture longitudinal trends. Longitudinal studies are required to determine whether engagement, satisfaction, and clinical impact are maintained over time and to observe how user expectations and system performance evolve with continued use.

Fourth, all participants were recruited in South Korea. Although diabetes self-management practice and digital health adoption are broadly similar to those in many other high-income countries, cultural differences, such as perceptions of mobile services, dietary practices, and levels of digital literacy, may influence the applicability of generative AI chatbots in other populations [5,89]. Future studies may benefit from examining the use of generative AI chatbots across more diverse sociocultural and health care contexts.

### Conclusions

This study highlights the potential of generative AI chatbots to provide personalized guidance, trustworthy information, and holistic support for diabetes management. Both patients and specialists recognized these tools as valuable adjuncts to conventional health care, particularly for addressing routine inquiries, reinforcing self-management behaviors, and alleviating clinician workload. By demonstrating that a domain-specific generative AI chatbot grounded in verified medical sources can operate with transparency and minimal bias, this study offers a replicable model for safe AI deployment in health care.

Practical implications include guiding clinicians on integrating chatbots as complementary tools in chronic care workflows and informing developers on design strategies that enhance transparency, contextual relevance, and user trust. Future research would benefit from investigating the long-term impact of generative AI chatbots on clinical outcomes, explore integration with real-time biometric and EHR data, and evaluate their applicability across diverse chronic conditions and health care systems.

## Data Availability

The data generated and analyzed during this study are not publicly available due to participant privacy concerns, but may be shared by the corresponding author upon reasonable request.

## Appendix

Multimedia Appendix 1 Standards for Reporting Qualitative Research (SRQR) checklist.

Multimedia Appendix 2 Survey Items for Evaluating the Chatbot.

Multimedia Appendix 3 Screenshots and English translations of participants’ chat logs.

Multimedia Appendix 4 Top 10 Representative Questions for Each Theme.

## Abbreviations

AI: artificial intelligence
CGM: continuous glucose monitoring
EHR: electronic health record
RAG: retrieval-augmented generation
RQ: research question
