# Supplementary material for: Generative AI Chatbot for Diabetes Management: Formative 2-Part Qualitative Study Using DTalksBot Involving Patients and Clinicians
Source: JMIR Form Res. 2025 Nov 12;9:e72553. doi: 10.2196/72553 (PMC12658402; doi:10.2196/72553)
Supplement: Multimedia Appendix 2 [file formative_v9i1e72553_app2.docx]

All items were rated on a 5-point Likert scale (1 = Strongly disagree, 5 = Strongly agree).

Usability (System Usability Scale) [57]

1. I think that I would like to use this chatbot frequently.
2. I found the chatbot unnecessarily complex. (R)
3. I thought the chatbot was easy to use.
4. I think I would need technical support to use this chatbot. (R)
5. I found the various functions of the chatbot well integrated.
6. I thought there was too much inconsistency in the chatbot. (R)
7. I believe most people would learn to use this chatbot very quickly.
8. I found the chatbot very cumbersome to use. (R)
9. I felt very confident using the chatbot.
10. I needed to learn a lot before using the chatbot. (R)

Message Credibility [58]

1. The information provided by the chatbot is accurate.
2. The information provided by the chatbot is genuine.
3. The information provided by the chatbot is trustworthy.

Perceived Social Support [59]

1. This chatbot really tries to help me.
2. I received emotional support from the chatbot.
3. The chatbot provided genuine comfort.
4. I can rely on the chatbot when things go wrong.
5. I can talk to the chatbot about my problems.
6. I can share my joy and sorrow with the chatbot.
7. The chatbot values my feelings.
8. The chatbot helped me make decisions.

Empathy [60]

1. The chatbot seemed to understand how I feel.
2. The chatbot appeared to understand me.
3. The chatbot spoke on my behalf.

Trustworthiness [61]

1. The chatbot performs its support role well.
2. The chatbot is competent and skilled.
3. The chatbot is very knowledgeable.

Expectations [62]

1. Using the chatbot helped me understand diabetes better.
2. The chatbot helped me improve my health knowledge.

Satisfaction [63]

1. Communication with the chatbot was clear.
2. I could instantly understand what information the chatbot provides.
3. It felt like a continuous conversation.
4. The chatbot could understand the context.
5. The chatbot responded well even when my question was unclear.
6. The chatbot’s responses were easy to understand.
7. The chatbot understood what I wanted and helped me achieve it.
8. The chatbot provided the right amount of information.
9. The chatbot only gave me the information I needed.
10. The chatbot responded quickly.
