# Supplementary material for: Generative AI Chatbot for Diabetes Management: Formative 2-Part Qualitative Study Using DTalksBot Involving Patients and Clinicians
Source: JMIR Form Res. 2025 Nov 12;9:e72553. doi: 10.2196/72553 (PMC12658402; doi:10.2196/72553)
Supplement: Multimedia Appendix 3 [file formative_v9i1e72553_app3.docx]

| **1. Screenshots of P2’s chat logs** | **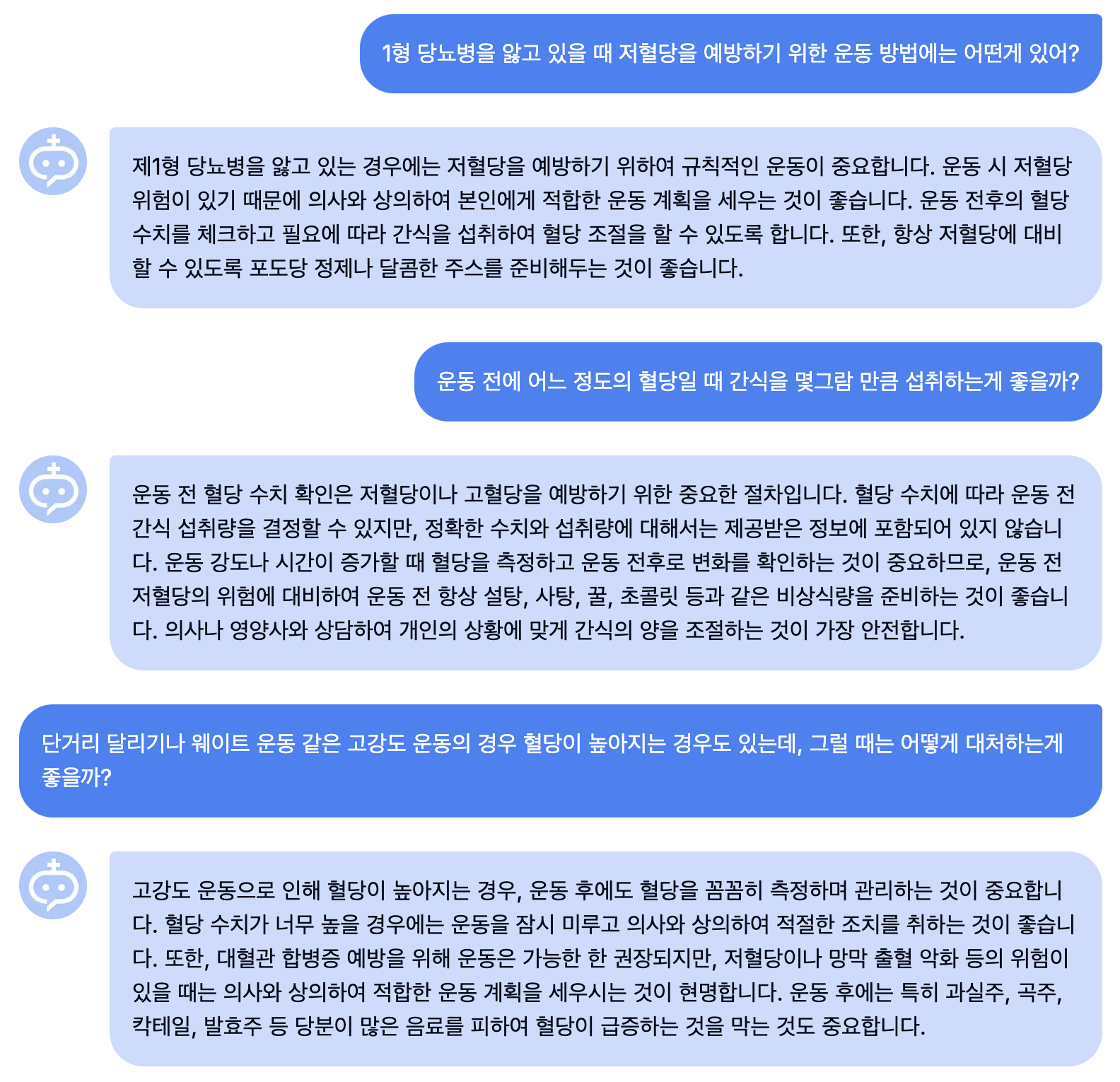** |
| --- | --- |
| **1.**  **English translations** | Q:  When you have type 1 diabetes, what kind of exercise can prevent hypoglycemia?  A (Chatbot):  When you have type 1 diabetes, regular exercise is important to prevent hypoglycemia. Since there is a risk of hypoglycemia during exercise, it is recommended to discuss with your doctor and establish a personalized exercise plan. It's also helpful to check your blood sugar levels before exercising and prepare snacks as needed to maintain your levels. To prevent sudden hypoglycemia, it’s good to have foods like grapes or sweet juice ready.  Q:  Before exercising, to what blood sugar level should I prepare snacks, and how much should I take?  A (Chatbot):  Checking your blood sugar level before exercise is important to prevent both hypoglycemia and hyperglycemia. Depending on your blood sugar level, you can determine whether to take snacks before exercising. However, there isn't a specific guideline for the exact number or amount. It’s important to monitor your blood sugar changes according to the intensity and duration of exercise. To prepare for the risk of hypoglycemia, it’s good to have simple snacks like candy, juice, or chocolate ready. It's safest to adjust the snack amount according to personal advice from your doctor or dietitian.  Q:  If I do short-distance running or walking, my blood sugar might go high. How should I handle that?  A (Chatbot):  If your blood sugar rises after high-intensity exercise, it is important to continue monitoring and managing it. If your blood sugar is too high, it may be necessary to stop exercising temporarily and consult your doctor for appropriate measures. Also, to prevent ketone build-up, it’s important to drink enough fluids and avoid sugary drinks like soda or juice if your blood sugar is high**.** |
| **2.**  **Screenshots of P7’s chat logs** | **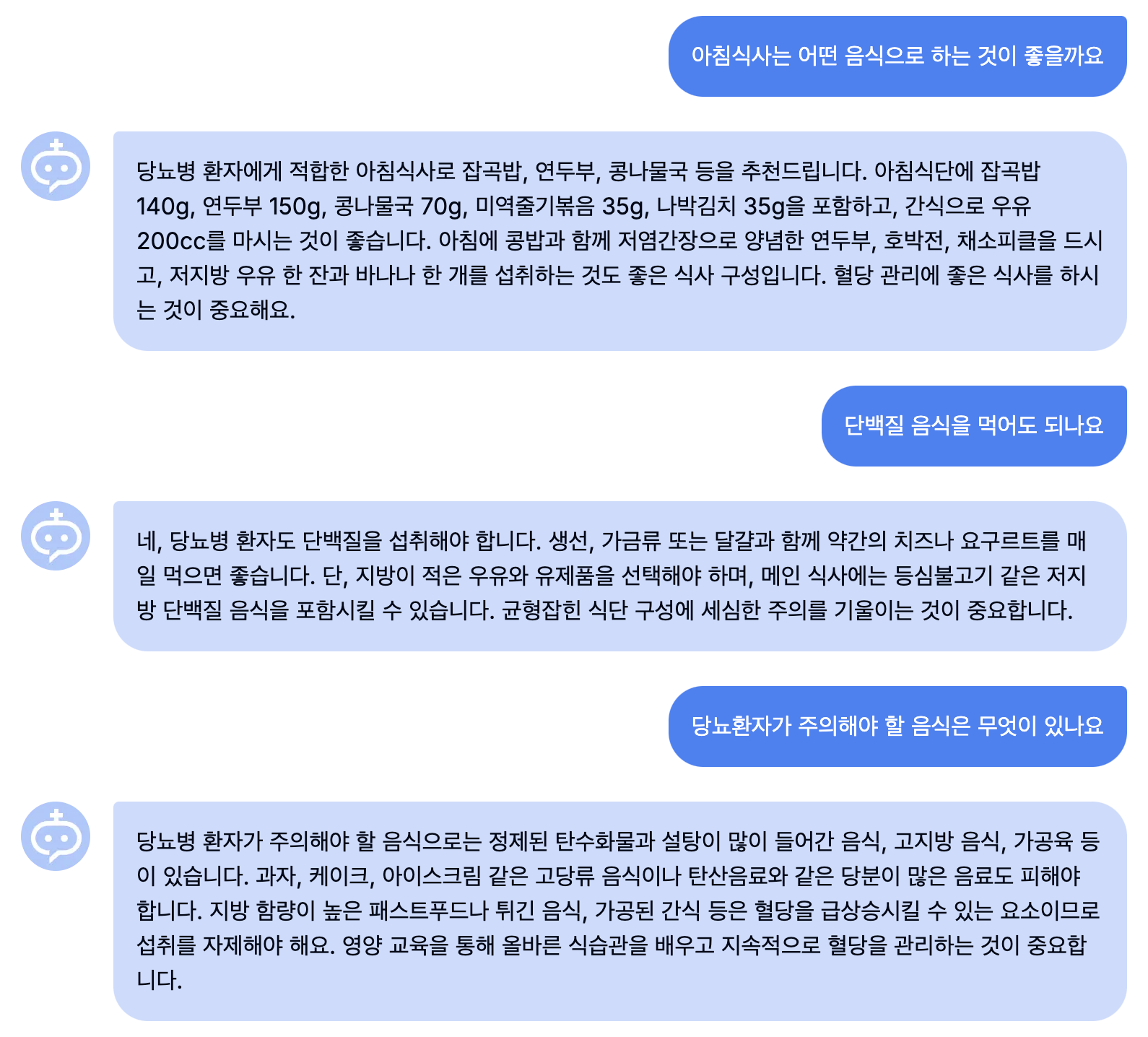** |
| **2.**  **English translations** | Q:  I have type 2 diabetes, and my biggest concern is complications. Is there a way to improve kidney function?  A (Chatbot):  Since you have type 2 diabetes and are worried about complications, particularly kidney complications, it’s important to control your blood sugar and blood pressure as optimally as possible. Monitoring your blood sugar continuously and maintaining a healthy diet and regular exercise are crucial. Also, it's important to adjust protein intake appropriately. In some cases, specific medications prescribed by your doctor may be needed. Consistency is key in health management, and I’m cheering you on for your continuous efforts and small successes. |
| **3.**  **Screenshots of P19’s chat logs** | **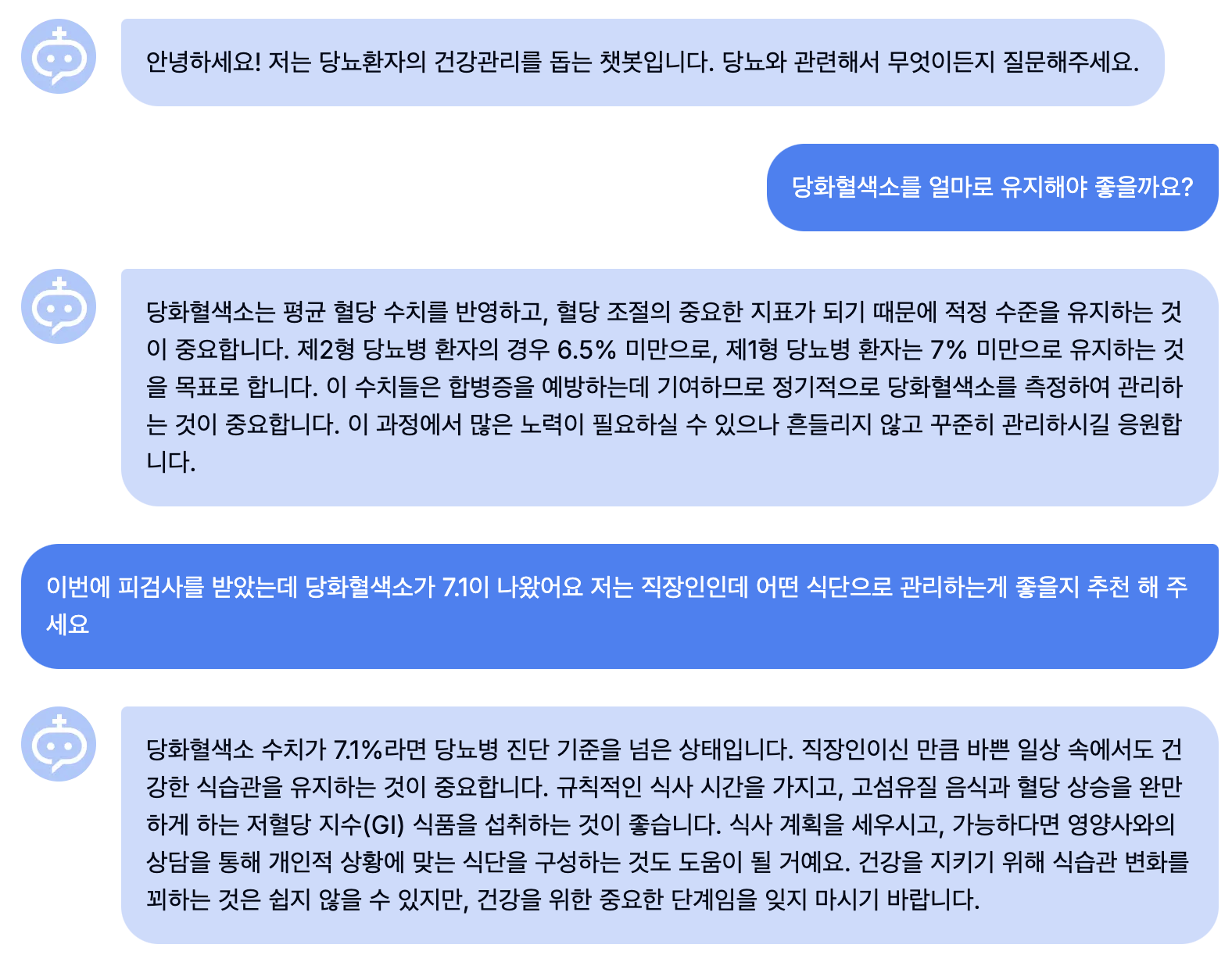**  **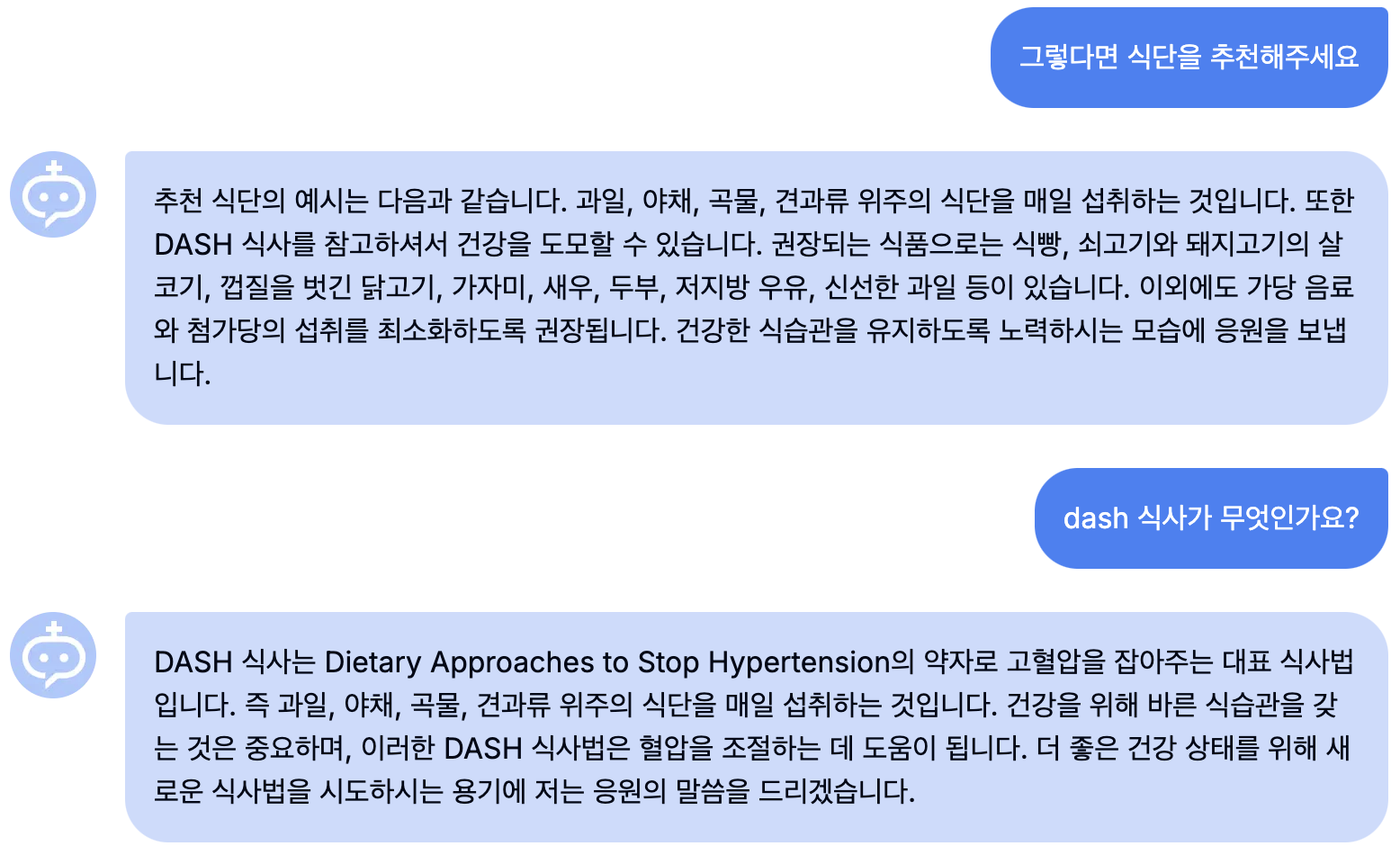**  **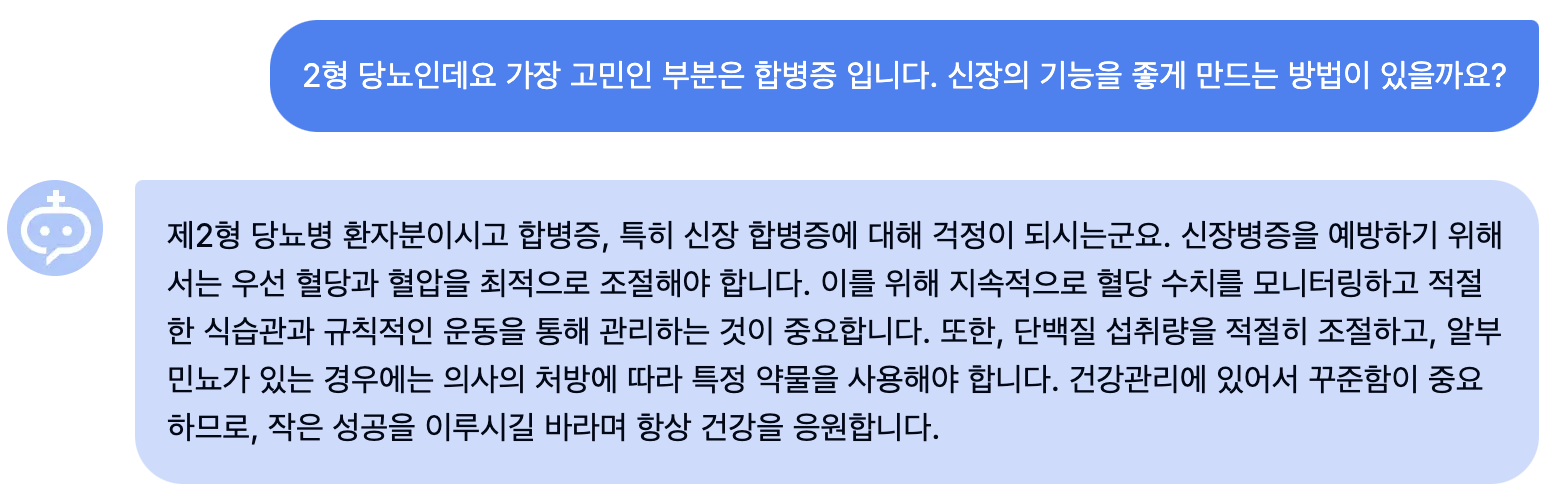** |
| **3.**  **English translations** | Chatbot:  Hello! I'm a chatbot supporting diabetes health management. Feel free to ask any diabetes-related questions.  Q:  What should my HbA1c level be?  A (Chatbot):  HbA1c reflects your average blood sugar levels and is an important marker for blood sugar control. For people with type 2 diabetes, it’s recommended to maintain an HbA1c below 6.5%, and for type 1 diabetes, below 7%. Regularly monitoring your HbA1c helps prevent complications. It may take effort, but stay consistent and I’m cheering for your steady management!  Q:  I got a blood test, and my HbA1c is 7.1%. I'm an office worker. Could you recommend a diet plan for me?  A (Chatbot):  An HbA1c level of 7.1% exceeds the diabetes diagnosis threshold. Since you're an office worker with a busy lifestyle, it's important to maintain healthy eating habits. Regular meal times and consuming low glycemic index (GI) foods that minimize blood sugar spikes are recommended. It's also good to consult with a dietitian to create a personalized diet plan. Remember that consistent dietary changes are important even if not easy.  Q:  Please recommend a diet plan.  A (Chatbot):  Here’s an example of a recommended diet: Eat a daily diet based on fruits, vegetables, grains, and nuts. Referring to the DASH diet can help with your health. Recommended foods include brown rice, lean beef or pork, skinless chicken, mackerel, shrimp, tofu, low-fat milk, and fresh fruits. It's also advised to minimize intake of sugary drinks and processed foods to maintain a healthy eating habit. Keep up your effort to maintain healthy habits!  Q:  What is the DASH diet?  A (Chatbot):  The DASH diet stands for "Dietary Approaches to Stop Hypertension," a diet designed to control high blood pressure. It emphasizes a daily intake of fruits, vegetables, grains, and nuts. Practicing the DASH diet can help manage blood pressure. I support your courage to adopt a healthier eating style for better health. |
| **4.**  **Screenshots of P12’s chat log** | 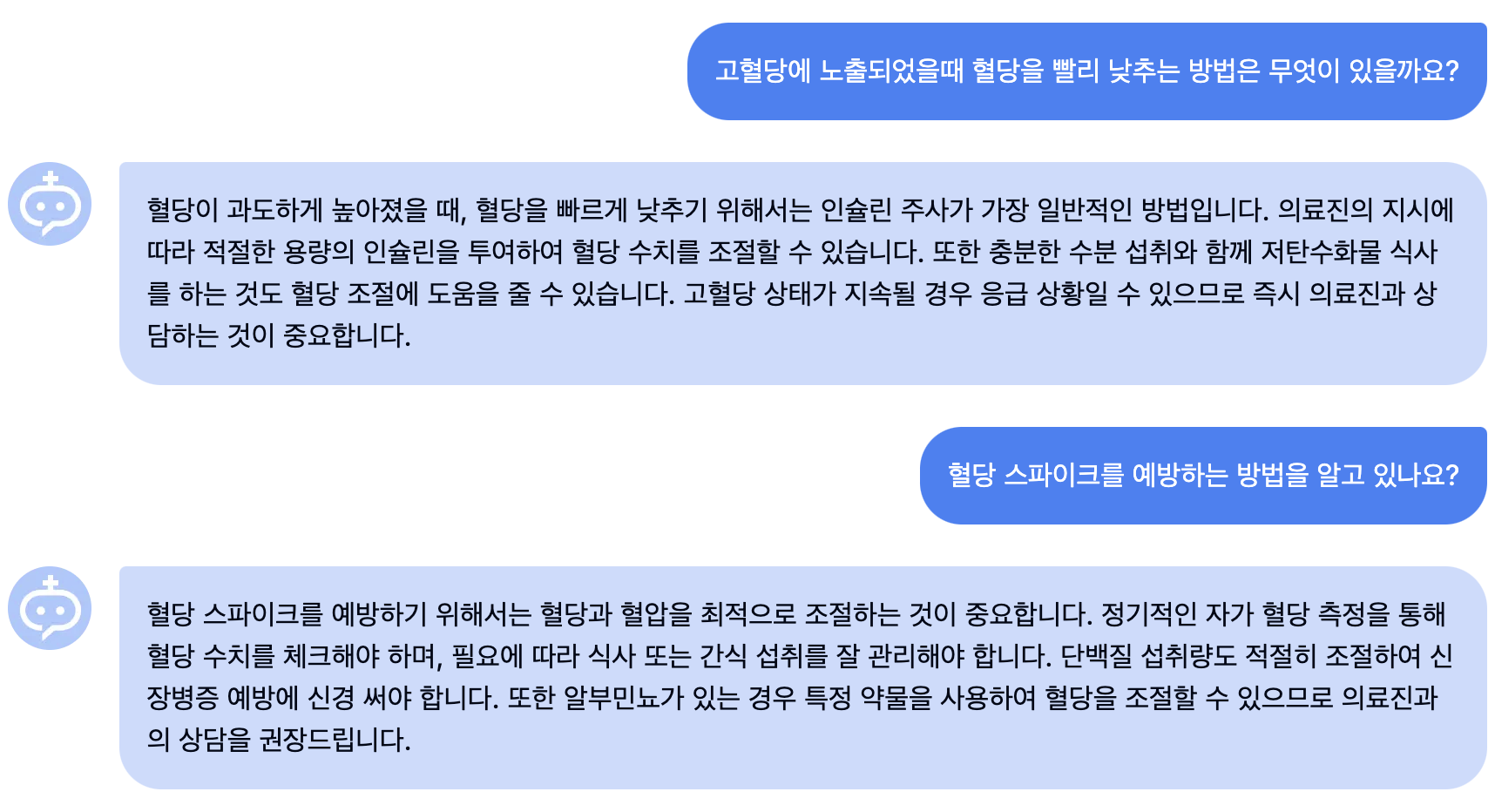 |
| **4.**  **English translations** | **Q:** When blood sugar levels are high, is there a way to lower them quickly?  **A (Chatbot):** When blood sugar rises excessively, the most common method to quickly lower it is insulin injection. Under medical supervision, an appropriate dose of insulin can help regulate blood sugar levels. Additionally, drinking enough water and consuming a low-carbohydrate meal can aid in blood sugar control. If high blood sugar persists, it could become an emergency situation, so it is important to consult a healthcare provider immediately.  **Q:**  Do you know how to prevent blood sugar spikes?  **A (Chatbot):**  To prevent blood sugar spikes, it is important to optimally control both blood sugar and blood pressure. Regular self-monitoring of blood sugar levels is necessary, and dietary or snack intake should be properly managed when needed. Protein intake should also be adjusted appropriately to prevent complications. If you have albuminuria, specific medications may be required to control blood sugar, so consultation with a healthcare provider is recommended. |
| **5.**  **Screenshots of P14’s chat log** | 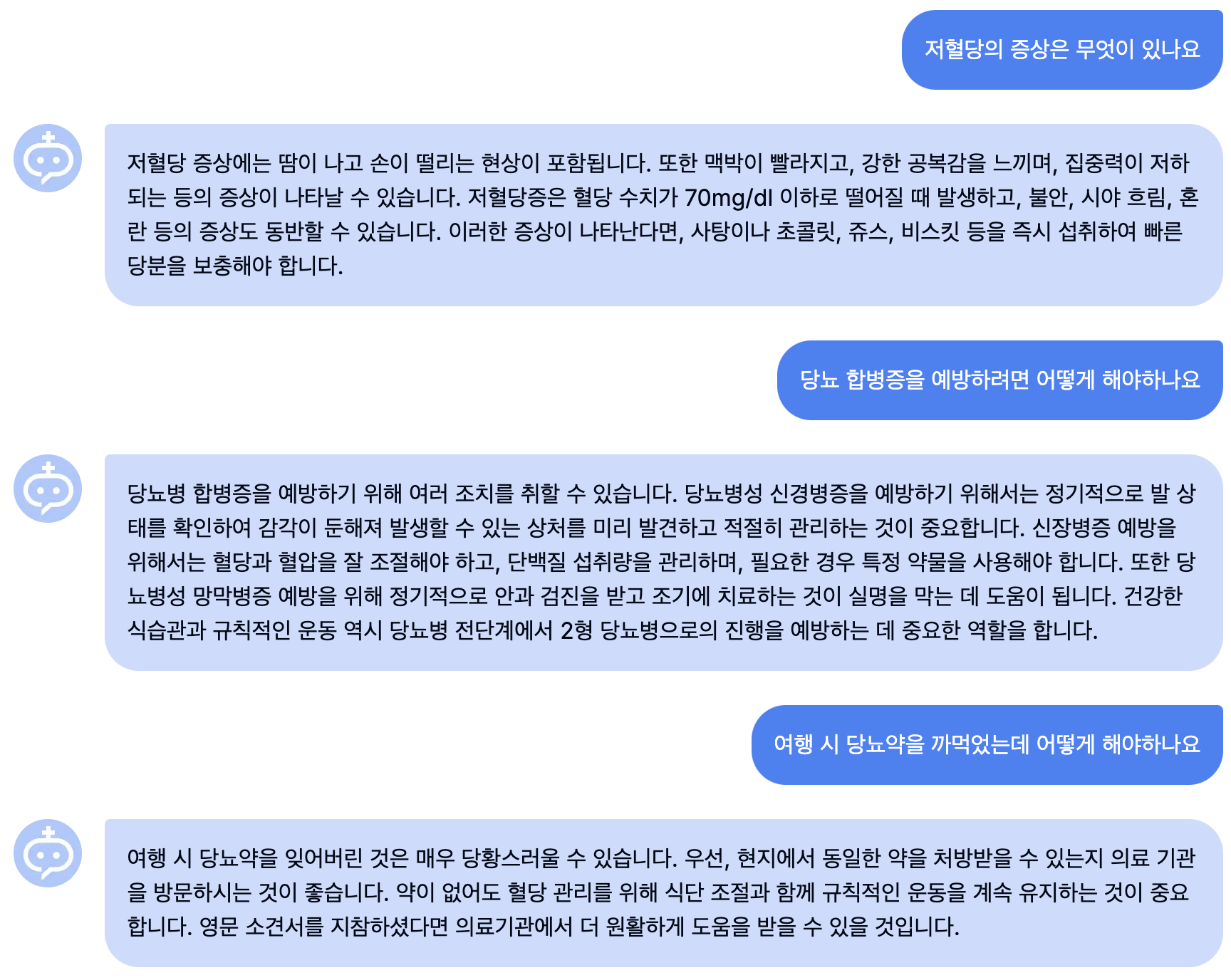 |
| **5.**  **English translations** | **Q:** What are the symptoms of hypoglycemia?  **A (Chatbot):** Symptoms of hypoglycemia can include sweating and trembling hands. Other symptoms may include a rapid heartbeat, intense hunger, feeling anxious, and decreased concentration. Hypoglycemia typically occurs when blood sugar drops below 70 mg/dL and may also cause symptoms like anxiety, blurred vision, dizziness, or confusion. If these symptoms appear, it is important to immediately consume foods like candy, chocolate, juice, or biscuits to quickly restore blood sugar levels.  **Q:** How can diabetic complications be prevented?  **A (Chatbot):** Several measures can be taken to prevent diabetic complications. To prevent diabetic neuropathy, it is important to regularly check for any injuries or changes on the skin and manage them appropriately. For kidney disease prevention, good blood sugar control and regulated protein intake are crucial, and in some cases, specific medications may be necessary. Regular eye exams are also important to prevent diabetic retinopathy and vision loss. Maintaining a healthy diet and regular exercise also help slow the progression of diabetes into more serious complications.  **Q:** What should I do if I forget to bring my diabetes medication while traveling?  **A (Chatbot):** Forgetting your diabetes medication during travel can be very concerning. First, visit a local medical facility to obtain a prescription for the same medication if possible. If that’s not possible, immediately consult a healthcare provider for advice on how to adjust your lifestyle and manage your blood sugar levels. It’s helpful to carry a list of your medications and medical conditions when traveling so that you can receive assistance more quickly if needed. |
| **6.**  **Screenshots of P18’s chat log** | 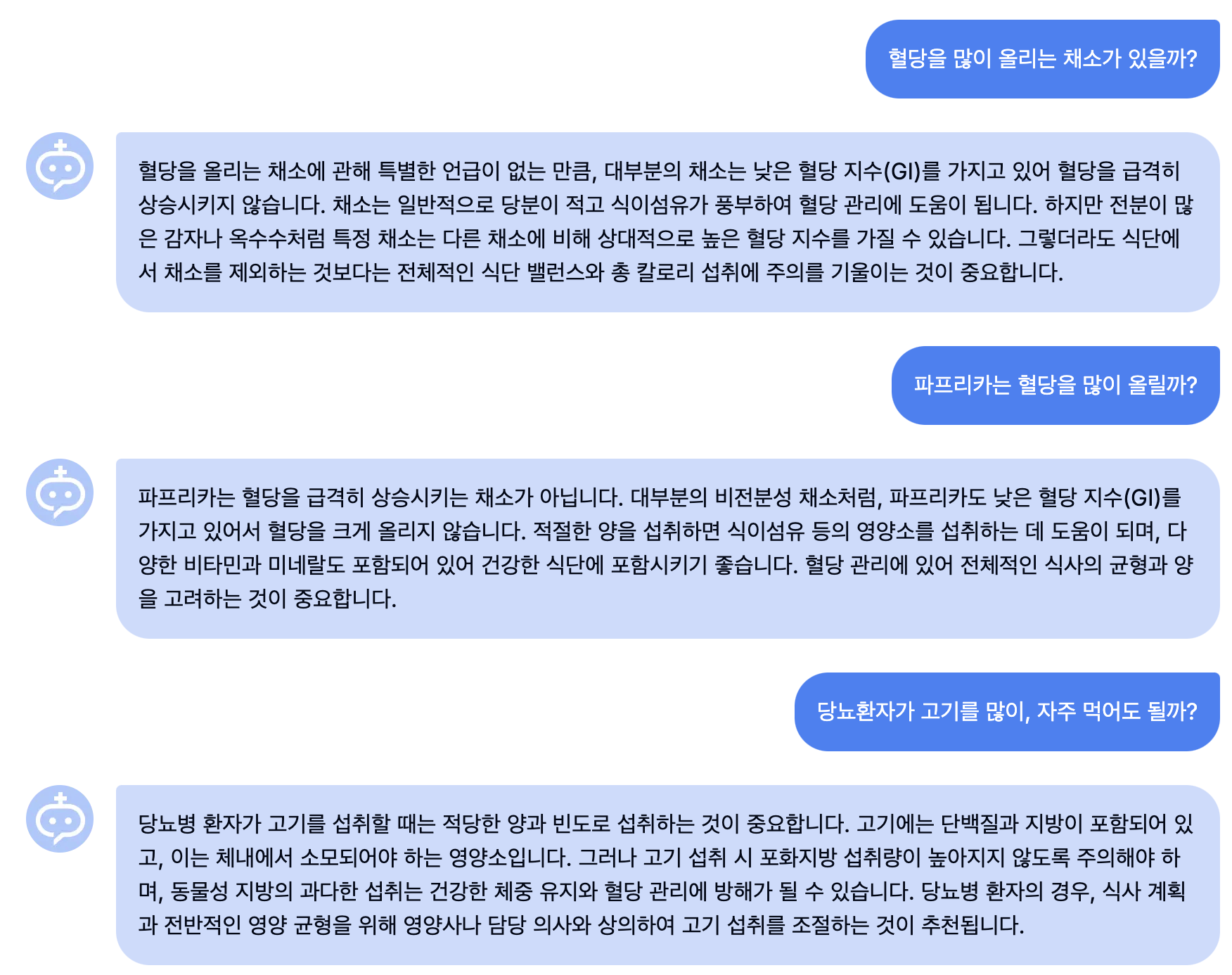 |
| **6.**  **English translations** | **Q:** Are there any vegetables that raise blood sugar significantly?  **A (Chatbot):** Since there are no specific vegetables known to significantly raise blood sugar, most vegetables have a low glycemic index (GI) and do not rapidly increase blood sugar levels. Vegetables are generally low in sugar and high in fiber, which helps control blood sugar. However, certain starchy vegetables like potatoes and corn can have relatively higher glycemic indices compared to other vegetables. Instead of avoiding specific vegetables, it’s more important to be mindful of the overall calorie intake and balance of your entire meal.  **Q:** Does bell pepper raise blood sugar a lot?  **A (Chatbot):** Bell peppers are not vegetables that rapidly raise blood sugar. Like most non-starchy vegetables, bell peppers have a low glycemic index (GI) and do not significantly raise blood sugar levels. They are also rich in vitamins and minerals, making them a healthy addition to your diet. It’s good to consider the overall balance and nutritional value of your diet rather than worrying about blood sugar increases when eating bell peppers.  **Q:** Can diabetics eat meat frequently?  **A (Chatbot):** It’s important for diabetics to consume an appropriate amount of meat, but not excessively. Meat is a good source of protein and helps maintain muscle mass and body functions. However, it’s better to choose lean meats and limit the intake of fatty meats to maintain overall health. Excessive consumption of animal fats can have a negative impact on health. It’s recommended to consume lean cuts of meat and balance the meal with plenty of vegetables and healthy carbohydrates to support blood sugar control. |
| **7.**  **Screenshots of P20’s chat log** | 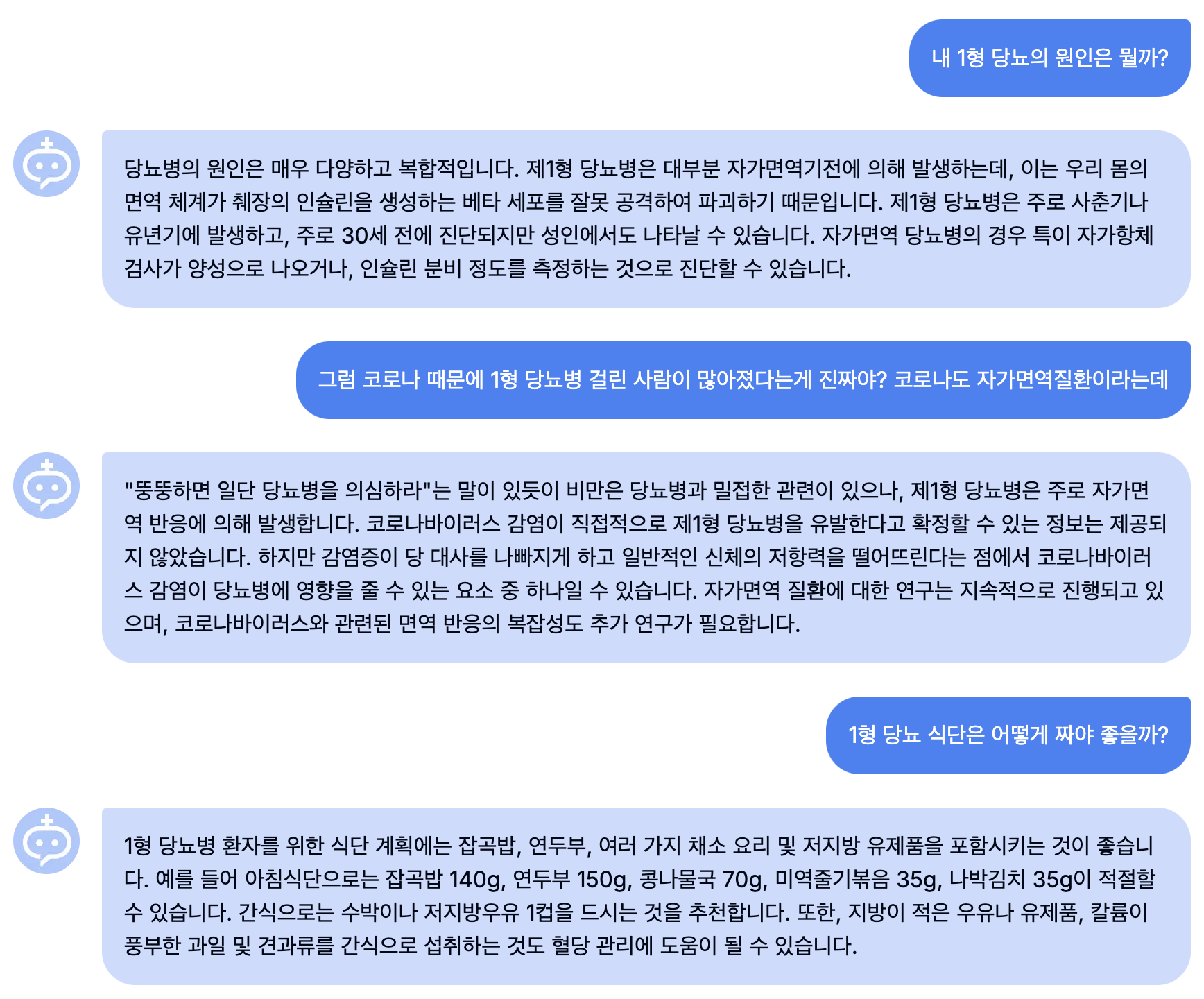  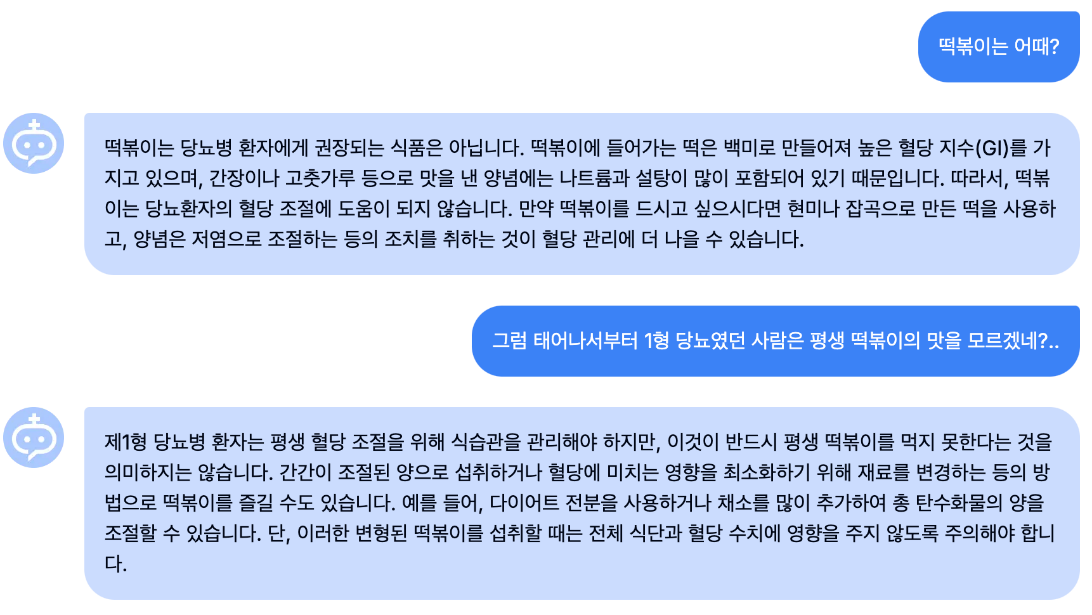 |
| **7.**  **English translations** | **Q:** What causes type 1 diabetes?  **A (Chatbot):** The causes of diabetes are very diverse and complex. Type 1 diabetes is mostly caused by an autoimmune reaction, where the body’s immune system mistakenly attacks the beta cells in the pancreas that produce insulin. Type 1 diabetes usually occurs during childhood or adolescence but can also appear in adults. In autoimmune diabetes, diagnosis is often made by detecting specific autoantibodies or by measuring insulin production levels.  **Q:** Is it true that more people developed type 1 diabetes because of COVID-19? I heard COVID-19 is also an autoimmune disease.  **A (Chatbot):** There is a saying, "Be alert for diabetes after COVID-19," and while there is some association, type 1 diabetes mainly results from autoimmune reactions. There is currently no confirmed evidence that COVID-19 infection directly causes type 1 diabetes. However, infections can worsen metabolic stress and impair general immune function, which may indirectly influence diabetes risk. Research on the link between autoimmune diseases and COVID-19 is ongoing, and further studies are needed.  **Q:** How should I plan meals for type 1 diabetes?  **A (Chatbot):** Meal planning for type 1 diabetes should include brown rice, tofu, various vegetable dishes, and low-fat dairy products. For example, a breakfast meal might include 140g of brown rice, 150g of tofu, 70g of soybean soup, 35g of stir-fried vegetables, and 35g of seasoned seaweed. As snacks, it’s recommended to consume low-fat milk or yogurt, along with fruits and nuts, which can help with blood sugar management.  **Q:** What about tteokbokki (Korean spicy rice cakes)?  **A (Chatbot):** Tteokbokki is not recommended for people with diabetes. The rice cakes in tteokbokki are made from white rice, which has a high glycemic index (GI), and the sauce often contains a lot of sugar and sodium from soy sauce or chili paste. Therefore, tteokbokki is not helpful for blood sugar control. If you still wish to eat tteokbokki, using brown rice or whole grain rice cakes and reducing the sodium content in the sauce would be better for blood sugar management.  **Q:** Then someone who had type 1 diabetes since birth would never know the taste of tteokbokki?..  **A (Chatbot):** People with type 1 diabetes must manage their diet for lifelong blood sugar control, but that doesn't mean they can never eat tteokbokki. If the sauce is modified to reduce sodium and sugar or if the ingredients are adjusted to minimize blood sugar impact, they can still enjoy tteokbokki. For example, they could use diet-friendly recipes or add a lot of vegetables to reduce the total carbohydrate load. However, even when eating modified tteokbokki, it’s important to monitor the overall meal composition and blood sugar levels carefully. |
| **8.**  **Screenshots of P22’s chat log** | 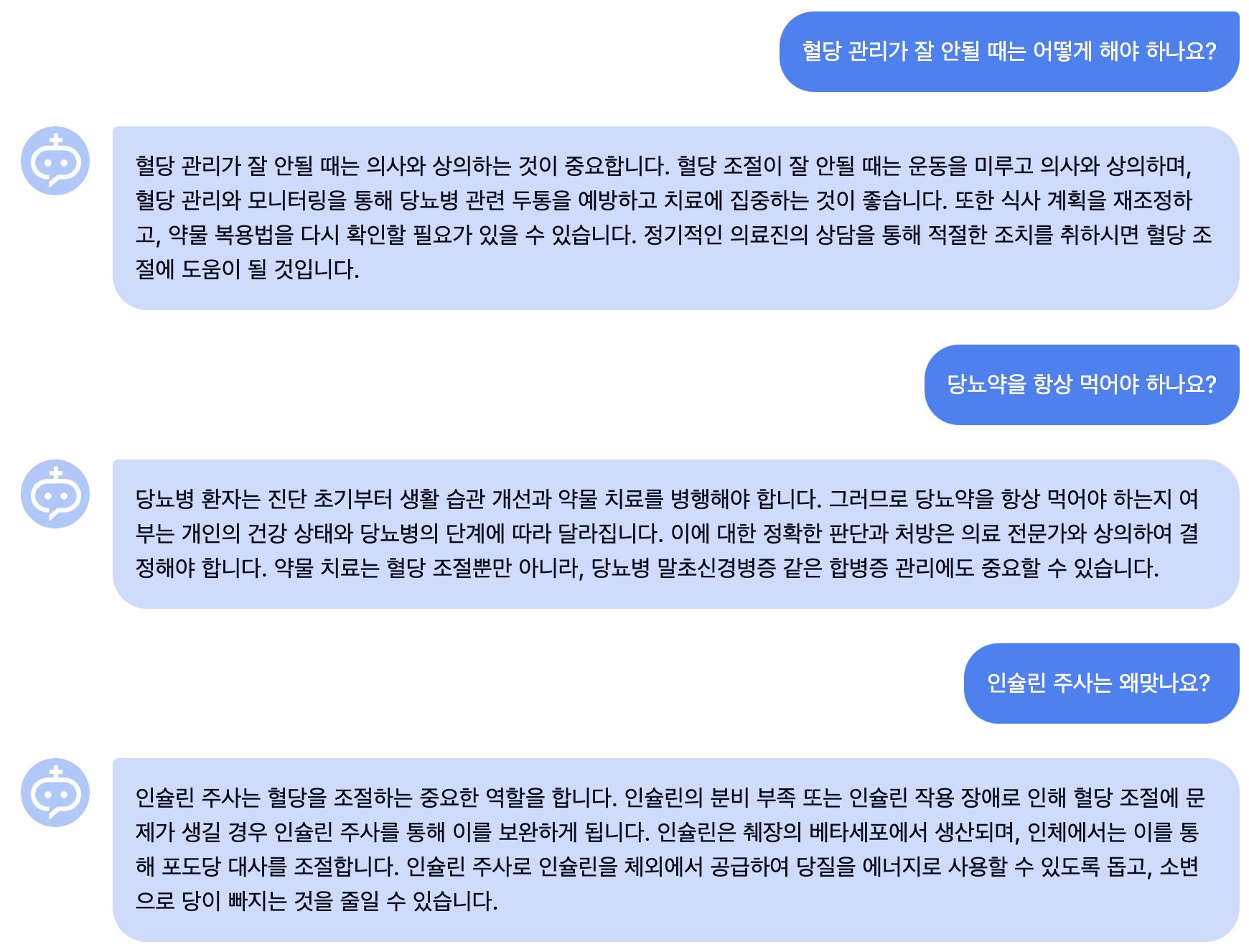 |
| **8.**  **English translations** | **Q:** What should I do when blood sugar control is poor?  **A (Chatbot):** When blood sugar control is poor, it is important to consult with your doctor. If blood sugar regulation is not going well, it's recommended to review and adjust your exercise plan with your doctor, and focus on blood sugar management and monitoring to prevent diabetes-related complications. It’s also good to reassess and adjust your meal plan. Reviewing your medication regimen may also be necessary. Regular medical consultations can help you take appropriate actions for better blood sugar control.  **Q:** Do I have to take diabetes medication all the time?  **A (Chatbot):** Diabetes patients should improve their lifestyle habits and take medication consistently from the time of diagnosis. However, whether diabetes medication needs to be taken continuously depends on the individual's health condition and the stage of diabetes. Therefore, a proper judgment should be made through consultation with a healthcare provider. Medication not only helps control blood sugar levels but also plays an important role in preventing diabetes-related complications such as hypertension and cardiovascular disease.  **Q:** Why are insulin injections necessary?  **A (Chatbot):** Insulin injections play an important role in controlling blood sugar. When the body's insulin production is insufficient or there is insulin resistance, insulin injections are needed to help regulate blood sugar. Insulin allows glucose to enter cells to be used as energy, thus preventing excessive sugar buildup in the blood. Insulin injections also help prevent complications like diabetic ketoacidosis, which can occur due to insulin deficiency. |
| **9.**  **Screenshots of P23’s chat log** | 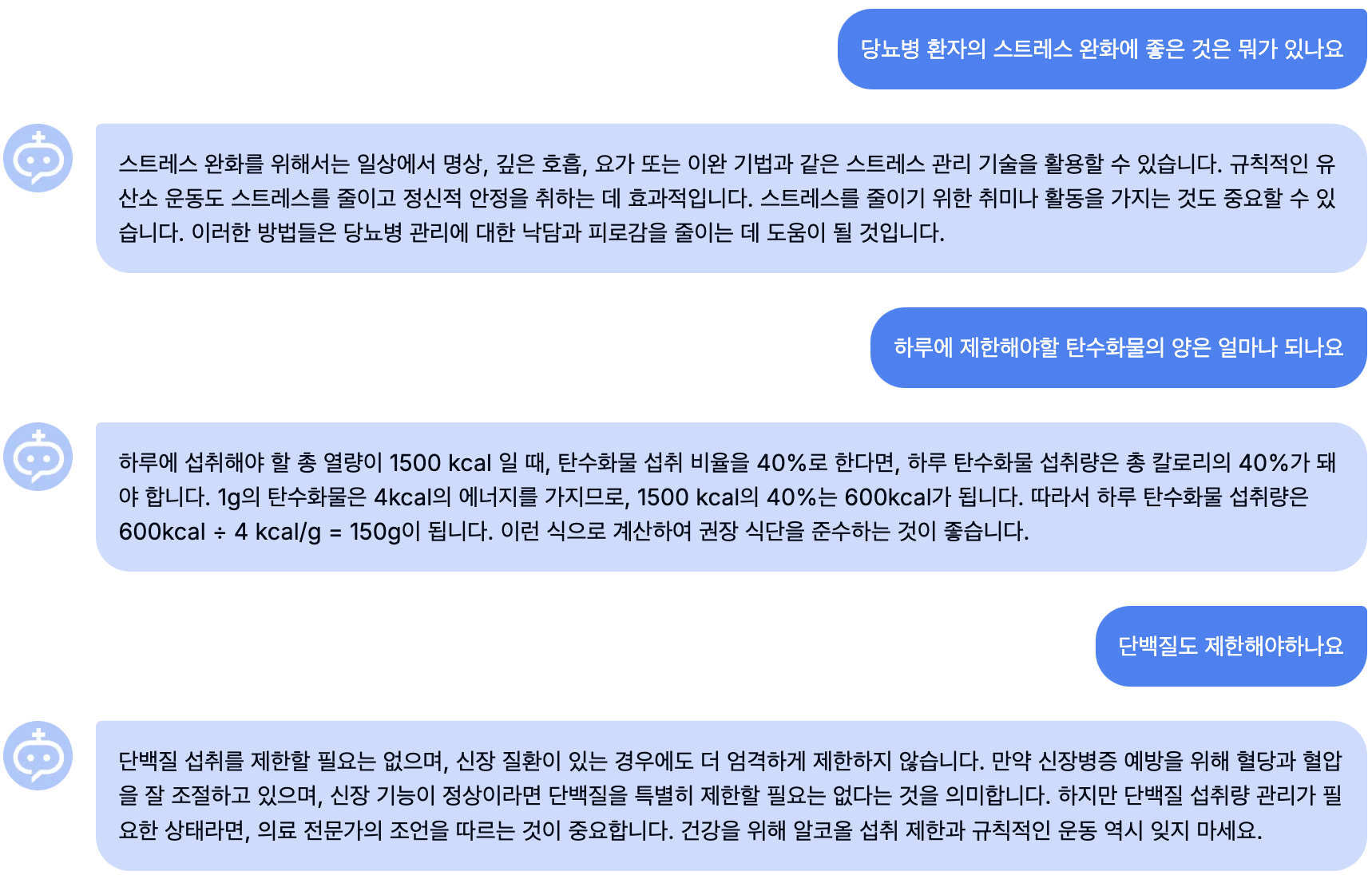 |
| **9.**  **English translations** | **Q:** What can help reduce stress for people with diabetes?  **A (Chatbot):** To relieve stress, you can use stress management techniques such as meditation, deep breathing, yoga, or relaxation methods in daily life. Regular aerobic exercise is also effective in reducing stress and achieving mental stability. Engaging in hobbies and activities aimed at reducing stress is important too. These methods can help lessen the psychological burden and fatigue associated with diabetes management.  **Q:** How much carbohydrate should be limited per day?  **A (Chatbot):** If your total daily calorie intake is 1500 kcal and carbohydrates should account for 40% of it, then the daily carbohydrate intake would be 40% of the total calories. Since 1 gram of carbohydrate provides 4 kcal of energy, 40% of 1500 kcal equals 600 kcal. Thus, the daily carbohydrate intake should be 600 kcal ÷ 4 kcal/g = 150g. It's important to calculate it this way and adhere to the recommended diet plan.  **Q:** Do I also need to limit protein intake?  **A (Chatbot):** There is generally no need to strictly limit protein intake, and even in cases of kidney disease, extreme restriction is not usually necessary. If your kidney function is normal, there's no special need to limit protein. However, if you are at risk of kidney problems, it’s important to follow your healthcare provider’s advice regarding protein intake. Also, don’t forget that limiting alcohol intake and maintaining regular exercise are important for overall health. |
| **10.**  **Screenshots of P5’s chat log** | 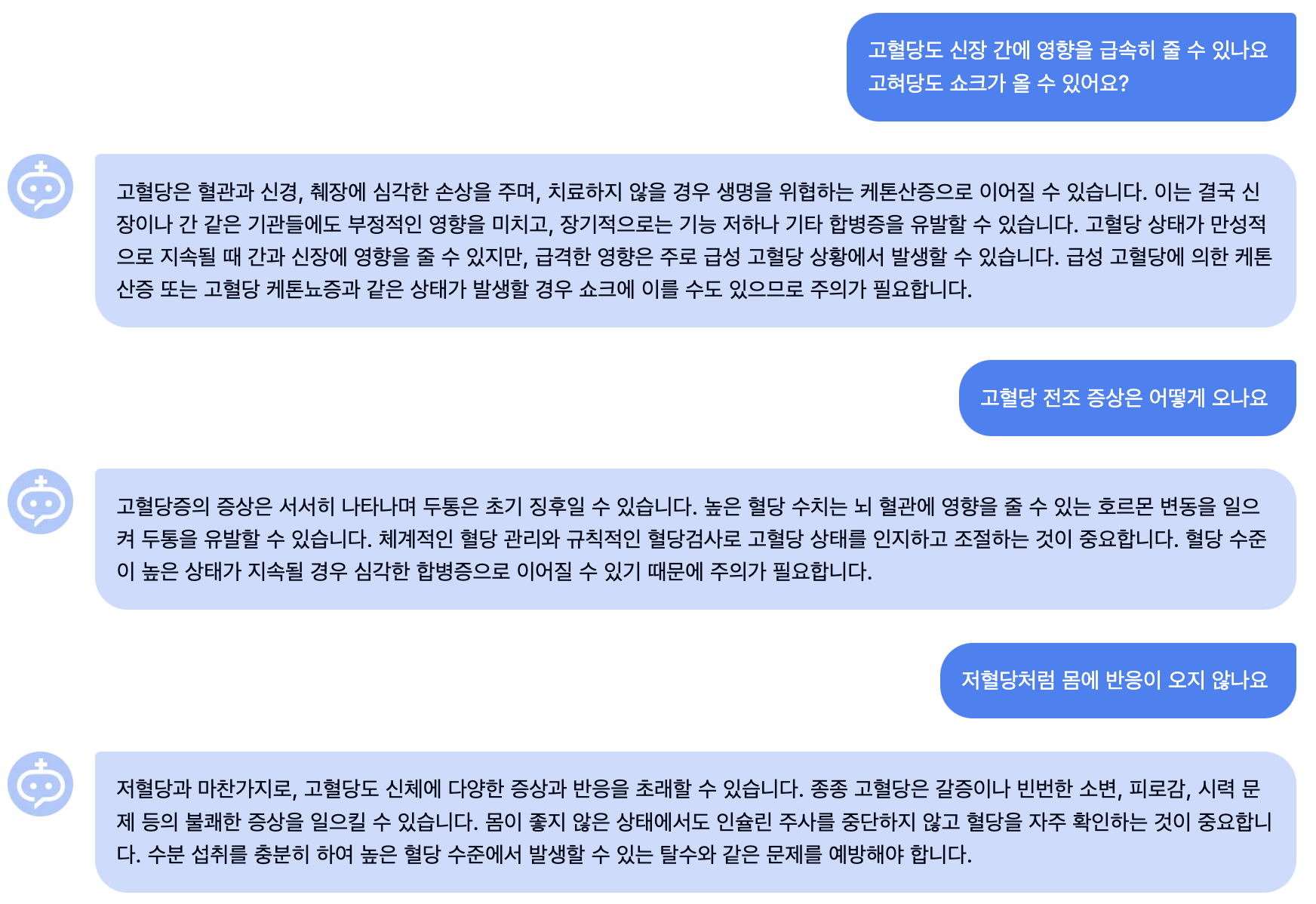 |
| **10.**  **English translations** | **Q:** Can hyperglycemia immediately affect the kidneys or liver? Can hyperglycemia cause shock?  **A (Chatbot):** Hyperglycemia can cause severe damage to blood vessels, nerves, and organs, and if left untreated, it can lead to life-threatening conditions such as ketoacidosis. It negatively affects various organs like the liver and kidneys over time and can trigger additional complications. If hyperglycemia persists long-term, it may gradually impair kidney function. However, acute effects typically occur in cases of severe hyperglycemia, which can cause complications such as ketoacidosis. Therefore, caution is necessary.  **Q:** How do the symptoms of hyperglycemia appear?  **A (Chatbot):** Symptoms of hyperglycemia appear gradually, and early symptoms may include headaches. High blood sugar levels can cause hormone imbalances that affect blood vessels in the brain, potentially leading to headaches. Recognizing and managing hyperglycemia early through systematic blood sugar monitoring is important. If high blood sugar levels persist, they can lead to serious complications, so careful management is necessary.  **Q:** Does the body react to hyperglycemia like it does to hypoglycemia?  **A (Chatbot):** Like hypoglycemia, hyperglycemia can cause a variety of symptoms and reactions in the body. Severe hyperglycemia may cause symptoms such as thirst, frequent urination, fatigue, blurred vision, and concentration difficulties. Even when feeling unwell, it’s important not to stop insulin injections without consulting a doctor and to monitor blood sugar levels frequently to prevent dehydration or other problems caused by high blood sugar. |
