# Supplementary material for: Generative AI Chatbot for Diabetes Management: Formative 2-Part Qualitative Study Using DTalksBot Involving Patients and Clinicians
Source: JMIR Form Res. 2025 Nov 12;9:e72553. doi: 10.2196/72553 (PMC12658402; doi:10.2196/72553)
Supplement: Multimedia Appendix 4 [file formative_v9i1e72553_app4.docx]

**Theme S1. Personalized Health Advice and Guidance**

- (P22): There is a lot of foam in my urine. Could this be caused by diabetes?

- (DTalksBot): If you notice a lot of foam in your urine, it may be due to a high protein content. In people with diabetes, the presence of protein in the urine is called proteinuria and can indicate impaired kidney function. Poorly controlled diabetes over a long period can damage the kidneys, increasing the likelihood of proteinuria. If foamy urine persists, it is important to consult a specialist to determine the exact cause and take appropriate action.

- (P10): Is it okay to eat fried chicken occasionally?

- (DTalksBot): Eating fried chicken occasionally is acceptable. However, similar to red meat, poultry should also be consumed in moderation. When choosing a chicken sandwich for breakfast, grilled chicken is preferable. Fried chicken is high in fat and sodium, so it should be enjoyed only in small amounts. It is important to maintain a balanced diet with appropriate nutrients for overall health.

- (P11): Sometimes, after exercising, my blood sugar doesn’t decrease but actually increases. Why does that happen?

- (DTalksBot): An increase in blood sugar after exercise can occur for several reasons. When exercise intensity or duration increases, the body may perceive it as stress and release more glucose into the bloodstream. Additionally, the body may raise blood sugar levels as a compensatory response to prevent hypoglycemia during exercise. If this happens frequently, it is important to consult your doctor to find strategies for better blood sugar control. Regularly monitoring your blood sugar before and after exercise is also highly recommended.

- (P4): I occasionally forget to take my diabetes medication. Is it okay to miss a dose once or twice?

- (DTalksBot): When treatment plans are changed or blood sugar control is unsatisfactory, it is recommended to measure HbA1c every 2–3 months, and at least twice a year if stable. Missing a dose once or twice may not cause major problems, but frequent omissions can negatively affect blood sugar control. If you often forget to take your medication, consider setting alarms or using apps to help you remember. Regular HbA1c tests and discussions with your doctor about your medication adherence are important for managing your diabetes.

- (P19): I recently had a blood test, and my HbA1c level came back at 7.1%. As an office worker, could you recommend a diet plan?

- (DTalksBot): An HbA1c level of 7.1% exceeds the diagnostic threshold for diabetes. As an office worker with a busy lifestyle, it is important to maintain healthy eating habits. Try to stick to regular mealtimes and choose foods high in fiber and those with a low glycemic index (GI) to minimize blood sugar spikes. Planning meals in advance and consulting with a dietitian to develop a personalized meal plan could also be beneficial. Although making dietary changes can be challenging, remember that it is a crucial step toward protecting your health.

- (P2): Sometimes after high-intensity exercises like sprinting or weightlifting, my blood sugar rises. How should I manage this?

- (DTalksBot): If your blood sugar rises after high-intensity exercise, it is important to carefully monitor and manage it after working out. If your blood sugar is excessively high, it is advisable to postpone further exercise and consult your doctor for appropriate measures. While exercise is generally encouraged to prevent macrovascular complications, if there is a risk of hypoglycemia or worsening of conditions like retinal hemorrhage, you should work with your doctor to develop a suitable exercise plan. After exercising, it is also important to avoid sugary alcoholic beverages such as fruit wines, sake, cocktails, or fermented liquors to prevent rapid blood sugar spikes.

- (P14): I accidentally used insulin that was past its expiration date. What should I do?

- (DTalksBot): Using expired insulin may impact effective blood sugar control, as expired insulin might not work as intended. You should obtain and use a new supply of insulin. Additionally, frequently monitor your blood sugar to detect any irregularities. It is highly recommended to consult a healthcare professional or visit a medical facility promptly for advice.

- (P6): I like foods like tteokbokki (spicy rice cakes). How often can I eat it while preventing diabetes?

- (DTalksBot): Foods like tteokbokki are high in carbohydrates, so it is advisable for people at risk of diabetes to limit their intake. However, having a small amount occasionally is acceptable—about once a week might be appropriate. Since optimal quantity and frequency can vary depending on individual blood sugar control, it is best to consult a healthcare professional for personalized advice. Thank you for your efforts to maintain healthy eating habits, and please consider working with a dietitian or diabetes specialist to create a customized meal plan.

- (P15): When I experience hypoglycemia, should I drink orange juice or sugary drinks to quickly raise my blood sugar?

- (DTalksBot): When hypoglycemia symptoms occur, rapid sugar intake is necessary. Orange juice or sugary drinks can quickly raise blood sugar levels and are helpful in such situations. Alternatively, taking a tablespoon of sugar or honey, or eating three to four pieces of candy, is also recommended. While fruits contain natural sugars that can raise blood sugar, eating whole fruits like apples or oranges—rich in fiber—may help maintain more stable blood sugar levels compared to fruit juices.

- (P8): Sometimes after exercising, I feel dizzy. Could this be related to diabetic complications? What can I do about it?

- (DTalksBot): Dizziness after exercise can have multiple causes, and fluctuations in blood sugar are one possible reason. Exercise generally helps with blood sugar control, but it can sometimes cause hypoglycemia, leading to dizziness. It is important to manage your blood sugar before and after exercise and ensure it is not too low before you begin. Staying well-hydrated and maintaining consistent exercise routines are also crucial. If dizziness persists, consult a healthcare professional to adjust your exercise intensity and method according to your health condition. Taking these steps is important to ensure a healthy lifestyle.

**Theme S2. Concerns about Complications and Comorbidities**

- (P20): I heard that high blood sugar can damage peripheral nerves. Could that be why my fingers feel numb?

- (DTalksBot): Persistent high blood sugar can indeed damage the nervous system, leading to peripheral neuropathy. Numbness in the hands and feet is one of the common symptoms of peripheral neuropathy, and numbness in the fingers may be related. However, finger numbness is not exclusively caused by high blood sugar. It is important to consult a healthcare professional to determine the exact cause. For those managing diabetes, monitoring blood sugar levels and seeking additional testing or treatment when control is poor is highly recommended.

- (P4): Can you tell me what types of tests are used to detect diabetes complications?

- (DTalksBot): Several tests are used to detect diabetes complications. Screening for diabetes includes fasting plasma glucose tests, oral glucose tolerance tests, and HbA1c measurements. To prevent complications such as end-stage renal disease, tests such as urine protein/albumin tests and estimated glomerular filtration rate (eGFR) measurements are necessary and should be conducted immediately after diagnosis and monitored at least annually. Regular foot exams are also important for early detection of sensory neuropathy.

- (P2): How often should I get regular check-ups to manage diabetes complications?

- (DTalksBot): Regular check-ups are critical for managing diabetes complications. Strict control of blood sugar and blood pressure is necessary for managing diabetic retinopathy, and regular ophthalmologic exams are essential. Screening for peripheral and autonomic neuropathy should be performed routinely, and a comprehensive foot examination should be conducted at least once a year for all patients with diabetes. Monitoring for changes in cognitive function and evaluating patients at high risk for hypoglycemia should also be prioritized. The exact frequency of these examinations should be determined in consultation with your healthcare provider. All assessments should begin immediately after diagnosis to ensure effective diabetes management.

- (P21): How can I prevent itching?

- (DTalksBot): Several measures can help prevent itching. It is important to avoid irritating fabrics like synthetic fibers and wool, and to change habits such as scratching. Using gentle cleansers on the skin and patting the skin dry after bathing—rather than rubbing it—can also help. These small changes in daily habits can significantly aid in preventing itching.

- (P14): How can I prevent diabetes complications?

- (DTalksBot): There are several steps you can take to prevent diabetes complications. For diabetic neuropathy, regularly checking the condition of your feet and managing any injuries early is important. For preventing diabetic nephropathy, it is essential to control blood sugar and blood pressure, manage protein intake, and use specific medications if necessary. Regular eye exams and early treatment can help prevent vision loss due to diabetic retinopathy. Healthy eating habits and regular physical activity are also crucial in preventing the progression from prediabetes to type 2 diabetes.

- (P17): I was diagnosed with hypertension first. Is there a relationship between hypertension and diabetes?

- (DTalksBot): There is a strong relationship between hypertension and diabetes. If you were diagnosed with hypertension first, you should be mindful of the increased risk of developing diabetes. In Korea, over 70% of kidney transplant recipients had hypertension as an underlying condition, and about 30–40% had diabetes. It is important to focus not only on blood sugar management but also on treating dyslipidemia and hypertension, along with lifestyle modifications, to prevent complications.

- (P9): What are the symptoms of hypoglycemia?

- (DTalksBot): Symptoms of hypoglycemia include sweating, trembling hands, rapid heartbeat, hunger, and decreased concentration. Other symptoms may include headache, anxiety, blurred vision, and confusion. Hypoglycemia generally occurs when blood sugar drops below 70 mg/dL. If you experience these symptoms, prompt action is necessary. Immediate intake of sugar—such as candy, chocolate, juice, or biscuits—is recommended.

- (P15): I've had diabetes for a long time, and I know it can burden the kidneys. However, my regular complication tests show no kidney issues. Should I still get additional kidney tests because I feel the urge to urinate frequently?

- (DTalksBot): Frequent urination can have multiple causes and is a common symptom among people with diabetes. Since diabetes is a major cause of end-stage renal disease, it is important to monitor urine protein/albumin levels and eGFR regularly. Even if routine complication tests indicate no kidney issues, if the symptom persists, it may be wise to undergo additional kidney function testing. If you notice new or concerning symptoms, do not hesitate to consult your doctor for appropriate evaluation and testing.

- (P23): What causes acute complications?

- (DTalksBot): Acute complications can be triggered by infections, discontinuation of insulin use, myocardial infarction, and other factors. Abrupt changes in insulin use or the presence of infections can significantly impact metabolism and lead to acute complications. Cardiovascular diseases like myocardial infarction also increase the risk. Prompt recognition and response to symptoms are crucial for managing acute complications effectively.

- (P8): I have hepatitis B. What should I be cautious about regarding diabetes and potential complications?

- (DTalksBot): If you have hepatitis B, special attention is needed to manage both liver function and diabetes. Since obesity is closely linked to diabetes, weight management is crucial. Hepatitis can also increase the risk of developing diabetes, so regular blood sugar and liver function tests are recommended. If severe infections occur alongside diabetes, it can complicate treatment and cause blood sugar levels to rise. Therefore, diligent blood sugar control and careful management and monitoring of hepatitis are essential for maintaining your health.

**Theme S3. Medication and Treatment Exploration**

- (P1): If I am diagnosed with diabetes, will I have to take medication for the rest of my life?

- (DTalksBot): Diabetes management typically involves both lifestyle modifications and medication from the time of diagnosis. While diabetes is a chronic condition that requires long-term management, not all patients will need lifelong medication. With successful lifestyle changes and good management, it may be possible to adjust, reduce, or even discontinue medications in some cases.

- (P21): My blood sugar was high all day. Should I inject more Tresiba?

- (DTalksBot): Adjusting insulin doses should be approached cautiously. Tresiba is a long-acting insulin designed for long-term blood sugar control rather than immediate correction. If your blood sugar remains high throughout the day, it is important to identify the underlying cause rather than simply administering more insulin. Consultation with your doctor to assess the situation and determine the appropriate course of treatment is recommended.

- (P5): I heard that long-term use of diabetes medications can affect the kidneys and liver. Does insulin also have long-term effects?

- (DTalksBot): The impact of diabetes medications on the kidneys and liver can vary depending on the type of medication and the individual’s condition. Insulin itself, when used appropriately, generally does not directly harm the kidneys. However, uncontrolled diabetes increases the risk of kidney complications, making strict blood sugar management crucial. Regular consultations with your healthcare provider to monitor your blood sugar control are highly recommended.

- (P7): Can diabetes be managed through exercise and diet alone, without medication?

- (DTalksBot): Exercise and dietary improvements are crucial for managing diabetes and can significantly aid blood sugar control. However, lifestyle changes alone may not always be sufficient, and medication may be necessary depending on the individual’s health condition and disease progression. Regular medical consultations are important. Additionally, during extreme weather conditions or when blood sugar control is unstable, you should adjust your exercise routine in consultation with your doctor.

- (P8): If medication alone doesn’t control diabetes, what treatment options are available?

- (DTalksBot): If single-medication therapy does not achieve blood sugar targets, additional medications with different mechanisms of action may be added. Managing diabetes often requires combination therapy, alongside lifestyle measures such as regular exercise, diet management, and consistent blood sugar monitoring. Treatment plans, especially for patients with comorbid hypertension, should always be developed in consultation with healthcare providers.

- (P12): When using an insulin pump, is it still necessary to administer long-acting insulin?

- (DTalksBot): When using an insulin pump, rapid-acting insulin is typically administered continuously or at scheduled intervals, replacing the need for separate long-acting insulin. However, specific instructions and dosing plans should always be developed in consultation with a diabetes care specialist. Close collaboration with healthcare providers is essential for personalized treatment.

- (P15): I am confused because my doctor told me to take my diabetes medication after meals, but the pharmacist said before meals. What should I do?

- (DTalksBot): It is understandable to feel confused when receiving different instructions. Medication timing can vary depending on the specific drug and the patient’s condition. Both your doctor and pharmacist are considering your situation to recommend the most effective timing. It is important to clarify this discrepancy through direct communication with your healthcare team to ensure effective symptom management and to prevent complications. Please consult your physician again to confirm the appropriate timing for your medication.

- (P19): I currently take oral diabetes medications but want to reduce or discontinue them. How can I work towards that?

- (DTalksBot): If you wish to reduce or discontinue medication, it must be carefully managed under medical supervision. In type 2 diabetes, treatment often involves both oral medications and injectables. Achieving optimal blood sugar and blood pressure control through healthy eating, regular exercise, and close collaboration with your healthcare team can help you work toward medication adjustment while maintaining your quality of life.

- (P23): Is it possible to completely cure or significantly improve type 2 diabetes?

- (DTalksBot): Type 2 diabetes develops gradually and often without symptoms, and it is the most common form of diabetes in Korea. While complete cure is difficult, it is possible to manage blood sugar effectively and reduce the risk of complications through healthy lifestyle habits and regular exercise. Treatment typically includes both oral medications and injectables, aiming to manage symptoms and slow disease progression. Early intervention during the prediabetes stage is especially important, and ongoing efforts are critical for successful management.

- (P24): How do medications differ depending on the severity of diabetes?

- (DTalksBot): Medications for diabetes vary depending on the type and severity of the disease. For type 1 diabetes, insulin injections are essential because the body produces little or no insulin. In early type 2 diabetes, lifestyle management with diet and exercise may suffice, but medications, including oral agents or injectables, may become necessary over time. Other forms, such as gestational diabetes or other specific types, require tailored treatments based on their underlying causes. It is always important to develop a personalized treatment plan in consultation with your healthcare provider.

**Theme S4. Mental Health Management and Support**

- (P10): Does stress worsen diabetes?

- (DTalksBot): Yes, stress can worsen diabetes. Stress related to frustration or fatigue in managing diabetes may lead to unhealthy habits and neglect of diabetes care. Stress can also cause anxiety, making management even more difficult. Persistent stress can increase insulin resistance and stimulate the secretion of cortisol, raising the risk of diseases such as diabetes. Therefore, effective stress management is very important.

- (P19): When I am stressed, I crave sweets. Can you recommend healthy ways for a person with diabetes to relieve stress?

- (DTalksBot): Craving sweets when stressed is very common. Since reducing stress is crucial for diabetes management, it is important to find healthy coping strategies. You might try yoga or meditation for mental relaxation. Engaging in hobbies or taking regular light walks can also help alleviate stress. If you feel overwhelmed or fatigued, consulting a professional can be beneficial. Prioritizing your health and managing stress appropriately is essential—please keep this in mind. We are cheering you on!

- (P8): I run my own business and experience a lot of stress. How can I manage it?

- (DTalksBot): Managing stress is critical not only for mental health but also for diabetes management. Stress and anxiety can be alleviated through exercise, relaxation techniques, social support, and self-care. Regular exercise, meditation, or yoga can help. Talking with close friends or family members can also be effective in relieving stress. You are doing important work, and we encourage you to find healthy ways to cope with stress.

- (P11): How does stress affect blood sugar?

- (DTalksBot): Stress can negatively affect blood sugar control. Psychological stress can trigger physical reactions, such as skin irritation, impacting daily life. Stress related to diabetes may lead to frustration and fatigue, which in turn can result in unhealthy habits or neglect of diabetes management. Therefore, it is important to manage stress and anxiety through exercise, relaxation techniques, social support, and self-care.

- (P12): What should I do if I don't get enough sleep?

- (DTalksBot): When experiencing sleep deprivation, it is advisable to adjust the intensity and duration of exercise. If you are feeling particularly fatigued or unwell, opt for light activities such as walking rather than strenuous exercise. Resting and recovering your strength with adequate sleep before resuming regular exercise is important. It is also wise to adjust your daily schedule to ensure you can get sufficient sleep.

- (P17): Could stress be a cause of symptoms like itching?

- (DTalksBot): Yes, stress can be one of the causes of itching. Managing stress is essential for both mental and physical health. Chronic stress can increase cortisol secretion, weaken immunity, and raise the risk of diseases like diabetes. Managing stress and anxiety effectively through exercise, relaxation techniques, social support, and self-care helps maintain a healthy body and mind, and improves immune function.

- (P18): When feeling stressed about type 1 diabetes, what can I do?

- (DTalksBot): Feeling stressed about managing type 1 diabetes is common. It is important to find coping strategies to deal with this stress. Chronic stress can lead to unhealthy behaviors, so it is beneficial to take appropriate rest and seek help from mental health professionals if needed. Celebrating small daily successes and maintaining a positive attitude are important to prevent neglect of diabetes management.

- (P21): How can I manage stress effectively?

- (DTalksBot): Stress can be managed effectively through exercise, relaxation techniques, social support, and self-care. Exercise is a highly effective way to relieve stress, and relaxation methods like meditation can help calm the mind. Communicating with family and friends for social support is also important. Finding and practicing your own stress management strategies can make a significant difference.

- (P21): What is the relationship between depression and blood sugar? (P21)

- (DTalksBot): Depression is a common mental health issue among people with diabetes and can negatively impact blood sugar management. Depression can impair self-care abilities, leading to poorer blood sugar control. Therefore, managing mental health is as important as managing blood sugar for maintaining overall health in people with diabetes.

- (P24): What are good ways to cope with stress?

- (DTalksBot): There are various ways to manage stress and anxiety. Regular exercise can reduce stress and improve mood. Relaxation techniques and meditation can also help achieve mental calmness. Additionally, receiving social support from friends or family can be very helpful. Learning and practicing self-management skills is important to handle stressful situations effectively.
